# KRas4B-PDE6δ complex stabilization by small molecules obtained by virtual screening affects Ras signaling in pancreatic cancer

**DOI:** 10.1186/s12885-018-5142-7

**Published:** 2018-12-29

**Authors:** Diana Casique-Aguirre, Paola Briseño-Díaz, Ponciano García-Gutiérrez, Claudia Haydée González-de la Rosa, Reyna Sara Quintero-Barceinas, Arturo Rojo-Domínguez, Irene Vergara, Luis Alberto Medina, José Correa-Basurto, Martiniano Bello, Rosaura Hernández-Rivas, María del RocioThompson-Bonilla, Miguel Vargas

**Affiliations:** 1 Departamento de Biomedicina Molecular, Centro de Investigación y de Estudios Avanzados del Instituto Politécnico Nacional (CINVESTAV-IPN), Av. I.P.N, 2508 México City, Mexico; 2 Departamento de Química, Universidad Autónoma Metropolitana. Unidad Iztapalapa, México City, Mexico; 3 Departamento de Ciencias Naturales, Universidad Autónoma Metropolitana. Unidad Cuajimalpa, México City, Mexico; 4 Instituto Nacional de Cancerología, Unidad de Investigación Biomédica en Cáncer, México City, Mexico; 5 Laboratorio de Modelado Molecular y Diseño de Fármacos de la Escuela Superior de Medicina, Instituto Politécnico Nacional, México City, Mexico; 6 Investigación Biomédica y Trasnacional, Laboratorio de Medicina Genómica, Hospital 1° de Octubre, ISSSTE, México City, Mexico

**Keywords:** KRas4B, KRas4BG12C, PDE6δ, PDAC, Pancreatic cancer, Inhibitors, Virtual screening

## Abstract

**Background:**

The GTPase KRas4B has been utilized as a principal target in the development of anticancer drugs. PDE6δ transports KRas4B to the plasma membrane, where it is released to activate various signaling pathways required for the initiation and maintenance of cancer. Therefore, identifying new small molecules that prevent activation of this GTPase by stabilizing the KRas4B-PDE6δ molecular complex is a practical strategy to fight against cancer.

**Methods:**

The crystal structure of the KRas4B-PDE6δ heterodimer was employed to locate possible specific binding sites at the protein-protein interface region. Virtual screening of Enamine-database compounds was performed on the located potential binding sites to identify ligands able to simultaneously bind to the KRas4B-PDE6δ heterodimer. A molecular dynamics approach was used to estimate the binding free-energy of the complex. Cell viability and apoptosis were measured by flow cytometry. G-LISA was used to measure Ras inactivation. Western blot was used to measure AKT and ERK activation. MIA PaCa-2 cells implanted subcutaneously into nude mice were treated with D14 or C22 and tumor volumes were recorded.

**Results:**

According to the binding affinity estimation, D14 and C22 stabilized the protein-protein interaction in the KRas4B-PDE6δ complex based on in vitro evaluation of the 38 compounds showing antineoplastic activity against pancreatic MIA PaCa-2 cancer cells. In this work, we further investigated the antineoplastic cellular properties of two of them, termed D14 and C22, which reduced the viability in the human pancreatic cancer cells lines MIA PaCa-2, PanC-1 and BxPC-3, but not in the normal pancreatic cell line hTERT-HPNE. Compounds D14 and C22 induced cellular death via apoptosis. D14 and C22 significantly decreased Ras-GTP activity by 33% in MIA PaCa-2 cells. Moreover, D14 decreased AKT phosphorylation by 70% and ERK phosphorylation by 51%, while compound C22 reduced AKT phosphorylation by 60% and ERK phosphorylation by 36%. In addition, compounds C22 and D14 significantly reduced tumor growth by 88.6 and 65.9%, respectively, in a mouse xenograft model.

**Conclusions:**

We identified two promising compounds, D14 and C22, that might be useful as therapeutic drugs for pancreatic ductal adenocarcinoma treatment.

**Electronic supplementary material:**

The online version of this article (10.1186/s12885-018-5142-7) contains supplementary material, which is available to authorized users.

## Background

Pancreatic ductal adenocarcinoma (PDAC) is one of the most lethal malignant tumors, with a 5-year survival rate less than 6% [1]. To increase the survival rate of pancreatic cancer patients, it is necessary to search for improved tumor markers for earlier diagnosis and for new molecular targets for drug development. In most cases, PDAC is initiated by mutation (codon G12) of the KRas4B GTPase, which has been shown to drive pancreatic neoplasia. KRas4B plays a critical role in human cancer cell biology, with mutationally activated KRas4B shown in 95% of PDAC [2] cases. Mouse models in which the KRas4B oncogene can be switched on and off have impressively demonstrated that continuous oncogenic KRas4B signaling is essential for both the progression and maintenance of PDAC [3]. It has also become evident that sustained oncogenic KRas4B signaling is necessary for the growth and maintenance of metastatic lesions [4]. KRas4B transport to the PDE-modulated plasma membrane provides the opportunity to interfere with the Ras pathway. PDE6δ sustains the correct intracellular organization of KRas4B, and siRNA-mediated knockdown of PDE6δ leads to reduced Ras signaling and ERK phosphorylation [5]. The PDE6δ structure has a large hydrophobic cavity that accommodates the farnesyl group of Ras family GTPases. Some inhibitors have been developed to interfere with KRas4B localization. For example, Deltarasin at nanomolar concentrations disrupts the KRas4B-PDE6δ interaction and induces relocalization of Ras family proteins to endomembranes. This inhibitor perturbs the KRas4B-PDE6δ interaction, reduces proliferation and ERK1 phosphorylation in KRas4B-transformed pancreatic cancer cell lines, as well as tumor growth in xenografts of human pancreatic carcinoma cells [6]. However, since PDE6δ shuttles at least 37 other proteins [7], the function of other farnesylated proteins may be affected (http://www.innatedb.com/getGeneCard.do?id=83150) by Deltarasin. On the other hand, the farnesyl binding site of PDEδ is druggable, and Deltazinone 1, an analog of Deltarasin, has a higher affinity for the hydrophobic cavity of PDEδ [8]. However, this drug fails to improve the ability of Deltarasin to induce apoptosis and inhibit ERK phosphorylation in KRas4B-dependent cell lines. This effect is attributed to a more efficient displacement of Deltazinone 1 of PDE6δ by the activity of Arl2. Mice rapidly metabolize this compound, so it is not suitable for in vivo experiments [8]. Hence, it is necessary to search for new mechanisms and compounds that can affect the molecular mechanisms of KRas4B regulation by PDE6δ. Thus, we propose as a novel strategy the stabilization of the KRas4B-PDEδ complex for the treatment of PDAC. This strategy would prevent KRas4B from being released into the plasma membrane, thus inhibiting Ras signaling. The advantage of this strategy is that a small compound stabilizing the complex would exclusively recognize the KRas4B-PDE6δ heterodimer without affecting other molecules that are regulated by PDE6δ. Our goal was to find small molecules directed to the interface residues between KRas4B and PDE6δ through virtual screening in order to promote a more stable union between the targets of these molecules and evaluate their impact on KRas4B signaling in vitro and on a tumor model in vivo. We report here the structure-based discovery of two small molecules with high affinity for the KRas4B-PDE6δ complex, their impact on the KRas4B signaling pathway, and the tumor growth inhibition in xenografted mice.

## Methods

### Structure of the KRas4B-PDEδ complex

At the earliest stages of this work, the 3D structure of the KRas4B-PDEδ complex was unknown. Homology modeling of this complex was carried out using the Molecular Operating Environment package [9] employing as a template the previously reported RHEB-PDE6δ crystallographic structure (PDB ID 3T5G). A large set of structures was modeled resulting from different side-chain rotamers of newly incorporated residues. The structure with the best packing index was subjected to a global energy-minimization analysis with the CHARMM27 force field to yield the final model. Later on, two different crystallographic structures of the KRas4B-PDE6δ complex were reported and deposited at PDB ID: 5TAR and 5 TB5. They differ in the KRas4B C terminus close to the farnesylation site: the former shows an ordered structure, while the latter has a partially disordered segment. We used both, reporded 3D models and our model, the 5TAR and 5 TB5 structures to represent KRas4B-PDEδ intermolecular contacts and to guide the search for small organic compounds capable to simultaneously form interactions to both proteins, thus acting on the complex as molecular staples. Since in 3T5G structure RHEB is in contact with PDE6δ, we directly used solvent-exposed cavities composed of atoms from both proteins as targets for potential-ligand search. In the case of 5TAR, we used as targets the pockets close to the KRas4B-PDE6δ interface as found in the crystallographic lattice. The KRas4BG12C mutant corresponding to the predominant mutation present in MIA PaCa cell line was modeled with PyMOL v0.99 (https://pymol.org/2/).

### Virtual screenning

ENAMINE’s *Discovery Diversity Set* database (DDS) containing 50,240 low molecular weight compounds was selected for virtual screening. The 2D structures were translated into 3D structures using MOE-*Import Search*. Hydrogens and partial charges were assigned according to MMFF94 force field. Strong acids and bases are deprotonated and protonated, respectively. In order to simulate the molecular flexibility shown in real systems, structural conformers were constructed for each compound in DDS with MOE-Conformer Search and using a conformational energy cut-off of 3 kcal/mol with respect to the minimum energy conformer of each compound calculated according to the MMFF94 force field. The new database was then used for virtual screening. Potential binding sites, i.e. concave pockets at the protein-protein interface region in the KRas4B-PDE6δ model and crystalographic structures were identified with MOE-*SiteFinder* and CASTp server [10]. Previously, all crystallographic water and other organic molecules were removed. Hydrogen atoms and partial charges were added to the KRas4B-PDE6δ complex using the CHARMM27 force field. Virtual screening was carried out using MOE_*Dock* function and setting the *Alpha-Site-Triangle* and the *London dG* as the methods to bias the orientation search on potential binding sites and docking scoring function, respectively. At least 10,000 different orientations or poses on potential binding sites were proved and evaluated for each conformer, and the ten best coupling scores for each confomer were saved for further analysis. Finally, the KRas4B-PDEδ-ligand complexes with the best binding energies and frequencies were selected and evaluated with respect to the specific contacts of the compounds and the binding strengths, with preference given to the more polar compounds.

### Molecular dynamics (MD) simulations and binding free energy calculations

MD simulations of protein-protein and protein-ligand complexes were performed using AMBER 16 package [11] and the ff14SB forcefield [12]. Ligand charges for ligands and for no parameterized residues in proteins were determined using the AM1-BCC level and the general Amber force field (GAFF) [13]. For protein-protein and protein-ligand complexes a 15 Å and 12 Å, respectively, a rectangular-shaped box of TIP3P water model [14] was applied to solvate the complex and Cl^−^ and Na^+^ ions for protein-protein and protein-ligand systems were placed to neutralize the positive or negative charges around the complex models at pH 7. Before MD simulations, each molecular system was minimized through 3000 steps of steepest descent minimization followed by 3000 steps of conjugate gradient minimization. Then, systems were heated from 0 to 310 K during 500 ps (ps) of MD with restrained positions under an NVT ensemble. Next, MD simulations for 500 ps, in an isothermal-isobaric ensemble (NPT), were carried out to adjust the solvent density, followed by 600 ps of constant pressure equilibration at 310 K, using the SHAKE algorithm [15] on hydrogen atoms, and Langevin dynamics for temperature control. Equilibration runs were tailed by 100 ns-long MD simulations without position restraints, under periodic boundary conditions using an NPT ensemble at 310 K. The particle mesh Ewald method was utilized to describe the electrostatic term [16], and a 10 Å cut-off was used for the van der Waals interactions. Temperature and pressure were preserved using the weak-coupling algorithm [17] with coupling constants τT and τP of 1.0 and 0.2 ps, respectively. The time step of the MD simulations was set to 2.0 femtoseconds, and the SHAKE algorithm [15] was used to constrain bond lengths at their equilibrium values. Coordinates were saved for analyses every 50 ps. AmberTools14 was used to examine the time-dependence of the root mean squared deviation (RMSD), and the radius of gyration (RG), as well as for clustering analysis to identify the most populated conformation during the equilibrated simulation time.

### Calculation of binding free energies

Calculation of binding free energies was carried out using the MMGBSA approach [18–20] provided in the Amber16 suite [11]. 500 snapshots were chosen at time intervals of 100 ps from the last 50 ns of MD simulations, using a salt concentration of 0.1 M and the Generalized Born (GB) implicit solvent model [21]. The binding free energy of protein-protein and protein-ligand systems was determined as follows: ΔG_bind_ = G^complex^ – G^receptor^ – G^ligand^. ΔG_bind_ = ΔE_MM_ + ΔG_solvation_ – TΔS. ΔE_MM_ represents the total energy of the molecular mechanical force field that includes the electrostatic (ΔE_ele_) and van der Waals (ΔE_vdw_) interaction energies. ΔG_solvation_ signifies the desolvation free energy price upon complex formation, estimated from GB implicit model and solvent-accessible surface area (SASA) calculation that yield ΔG_ele/sol_ and ΔG_npol/sol_. Whilst, –TΔS is the solute entropy arising from structural changes that occur in the degrees of freedom of the free solutes and during formation of the protein-protein or protein-ligand complex.

### Reagents

Small organic compounds identified by virtual screening were purchased from ENAMINE (https://enamine.net/index.php?option=com_content&task=view&id=11) (Kyiv, Ukraine). The compounds were dissolved in 1.5% DMSO (SIGMA-ALDRIHC, catalog No. 276855-1 L). Deltarasin (hydrochloride) was purchased from Cayman Chemical (catalog No. 1440898–82-7).

### Cell culture

Human pancreatic cancer cell lines MIA PaCa-2, PanC-1, BxPC-3 and hTERT-HPNE were obtained from the American Type Culture Collection (ATCC; Manassas, VA). Cell lines were grown as monolayers in the specific medium suggested by ATCC.

### Cell viability assay

Cell lines were seeded at a density of 30,000 cells per well in a 96-well microtiter plate in growth medium and allowed to adhere for 24 h. Then, they were treated with 200 μM of each of the 38 compounds. Cell proliferation was assessed every 24 h during 3 days. Cell viability was determined by MTT (MTT Cell Proliferation Assay ATCC ^30-1010K^), by adding 10 μL of MTT per well, in dark conditions and incubated for 4 h. To solubilize the formazan crystals, 100 μL of acid isopropanol (50 mL of Triton X-100, 4 mL of HCl, 446 mL of isopropanol) was added, stirred continuously at room temperature and darkness for 3–4 h. The absorbance was measured in a spectrophotometer (Infinite F500 TECAN) at a wavelength of 570 nm. Each concentration was evaluated in triplicate, the solvent of the fractions and the untreated cells were taken as negative controls. The data are presented as the average percentage of proliferation and the standard deviation of the mean.

### IC50 determination

Cell lines were seeded at a density of 20,000 cells per well in a 96-well microtiter plate in growth medium and allowed to adhere for 24 h. Following the treatment with 200, 100, 50, 25, 12.5 and 6.25 μL of D14 and C22, respectively, cell viability was assessed for 5 days every 24 h. At the end of treatment, cell viability was determined by the CellTiter-Glo Luminescent Cell Viability Assay (Promega, catalog No. G7573). The dose-response curve was used to calculate the concentration of drug resulting in 50% inhibition of cell viability (IC50). The assays were repeated 5 times.

### Apoptosis assay

Approximately 5 X 10^5^ cells were seeded in 6-well plates for 24 h. Then, cells were treated with an IC_50_ concentration of D14 and C22 compounds and vehicle for 24 h. Cells were harvested with 0.25% trypsin, washed with phosphate buffered saline (PBS), and collected together by centrifugation. Apoptosis was determined using the Apoptosis/Necrosis Detection kit (Abcam, catalog No. ab176749, Cambridge, England) according to the manufacturer’s instructions and analyzed by flow cytometry using a FACSCalibur instrument (BD Biosciences), followed by data analysis using FlowJo software (Tree Star Inc). All experiments were performed in triplicate. Proteome Profiler Apoptosis Array (R&D Systems: ARY009) was used to evaluate the activity of D14 and C22 compounds on MIA-PaCa-2 cancer cells to determine the signaling pathways associated with cell death via Kras4B inhibition, which were done following the manufacturer’s instructions.

### Ras activation assay

The inactivation of Ras by D14 and C22 was determined using a G-LISA Ras activation assay kit (Cytoskeleton, catalog No. # BK131). The cells were serum-starved for 16 h and pre-treated with D14 and C22 at 99.3 μM and 137.5 μM, respectively, for 1 h; or Deltarasin at 5 μM for 3 h. Subsequently, the cells were stimulated with epidermal growth factor (EGF) (100 ng/mL) for 10 min. Lysates (1 mg/ml) were added to 96-well plates coated with Ras GTP-binding protein (Raf-RBD), following the manufacturer’s instructions. Experiments for each cell type were repeated three times.

### Western blot

The cells were serum-starved for 16 h and pre-treated with D14 at 99.3 μM or C22 at 137.5 μM for 1 h; or Deltarasin at 5 μM for 3 h. After pre-treatment, cells were stimulated with EGF at 100 ng/mL for 10 min*.* Whole-cell extracts were obtained by lysis of the Mia PaCa-2 cells in lysis buffer [20 mM Tris–HCl (pH 7.5), 1 mM EDTA, 150 mM NaCl, 1% Triton X-100, 1 mM NaVO_3_, 1 mM NaF, 10 mM β-glycerophosphate, 1 mM phenylmethylsulfonyl fluoride, and 1.2 mg/ml complete™ Lysis-M (Roche, Mannheim Germany) protease inhibitor cocktail]. The protein extracts were forced through a 22-gauge needle 10 times and centrifuged for 10 min at 14,000 rpm at 4 °C, and the protein concentration was determined using the Pierce™ BCA Protein Assay kit (Thermo Fisher Scientific, Waltham, MA, USA). Approximately 25 μg of protein was separated by 10% SDS-PAGE and transferred to nitrocellulose membranes. Then it was incubated with the following primary antibodies: Total ERK (Cell Signaling-9102; 1: 1000), pERK (Cell Signaling-9101; 1: 1000), Total AKT (Cell Signaling-9272 1: 1000), pAKT(Cell Signaling-4060 1: 1000), and anti-GAPDH (Gene Tex-GTX100118 1:100,000). Immunodetection was performed using a ChemiDoc™ Imaging Systems (BIO-RAD). Densitometry analysis was performed using the software ImageJ version 1.45 (National Institute of Health, USA).

### MAPK activation profiling

Cells were rinsed with cold PBS and immediately lysed in buffer supplemented with 4xcOmplete™ EDTA-free Ultra Protease Inhibitor Cocktail (Sigma-Aldrich) and 1xPhosSTOP™ (Sigma-Aldrich) at 4 °C for 30 min. Following centrifugation at 14,000×g for 5 min, supernatants were transferred into a clean tube and protein concentrations were determined using the Precision Red Advanced Protein Assay (Cytoskeleton, Inc. ADV02-A). Lysates were diluted and analyzed using the Human Phospho-MAPK Arrays (Proteome Profiler; R&D Systems; Minneapolis, MN, USA) according to the manufacturer’s instructions. Nitrocellulose membranes were scanned using a ChemiDoc™ Imaging Systems (BIO-RAD Laboratories, Inc.).

### Treatment of subcutaneous pancreatic carcinoma xenografts

Male immune-deficient Nu/Nu nude mice at 6 weeks of age (CINVESTAV, Mexico) were maintained in pathogen-free conditions with irradiated chow. The animals were subcutaneously injected in the back with 5 × 10^6^ MIA PaCa-2 cells per tumor in 0.1 ml of sterile phosphate-buffered saline. When MIA PaCa-2 cells reached palpable tumors (>100mm^3^), mice were divided randomly into three groups receiving vehicle (10% DMSO, 0,05% Carboxy Methyl Cellulose and 0,02% Tween 80 in PBS) (*n* = 10), D14 at 20 mg kg^− 1^ (*n* = 7), or C22 at 10 mg kg^− 1^ (*n* = 5 subcutaneous injected in the both flanks) and 20 mg kg^− 1^ (*n* = 10) administered by intra-peritoneal injection three times per week. Body weight was measured once a week, whereas tumors were measured twice weekly. Tumor sizes were calculated by the following formula: [(length x width^2^)/2 in mm.

### Histology and immunohistochemical staining of xenograft tumors

One day after the last treatment, mice were sacrificed in a CO_2_ chamber and the xenograft tumors were resected, fixed in 4% buffered formalin and embedded in paraffin. The tumors were cut using a microtome obtaining 2 μm slices. For hematoxylin and eosin (H & E) staining, the tissues were deparaffinized in xylene, hydrated in dehydrated alcohol starting from absolute ethanol to distilled water, stained for 2 min with Harris Hematoxylin, decolorized with 0.5% acid alcohol and fixing the color in lithium carbonate for 1 min, washed in distilled water, in 96% ethanol and stained with Sigma Eosin, washed and dehydrated in gradual alcohol changes until absolute alcohol was reached, allowed to dry at room temperature, mounted and observed, to identify the site of the injury. For immunohistochemical staining, the tissues were deparaffinized in xylene, hydrated in alcohols starting from absolute ethanol to distilled water, the epitopes were unmasked with 10 mM Citrate buffer at pH 6.03, washed with PBS pH 7.4. Endogenous peroxidase was blocked with 0.9% H_2_O_2_ for 15 min, then cross-sections were block with 3% BSA for 1 h. The antibodies Ki-67 (BIOCARE MEDICAL API 3156 AA) and CK 19 (GENETEX GTX110414) were diluted in PBS containing 1% BSA, the primary antibody was incubated at room temperature for 40 min, washed with PBS for 3 min, incubated with the biotinylated secondary antibody for 20 min at room temperature, washed with PBS for 3 min, incubated with streptavidin for 15 min, washed with PBS for 3 min. Reactions were incubated with 4% diaminobenzidine (DAB),counterstained with Harry’s Hematoxylin for 30 s, washed with distilled water, dehydrated in gradual changes of ethanol from distilled water to absolute Ethanol, allowed to dry at room temperature, mounted and observed.

### Statistical analysis

The statistical significances of the differences among the data were determined by Tukey’s multiple comparisons test, using GraphPad Prism® 6 software (San Diego, CA, USA). *P* < 0.05 was considered statistically significant. Values are presented as the means ± s.e.m. (standard error of the mean).

## Results

### The interface of KRas4B-PDE6δ complex

**Fig. 1:**
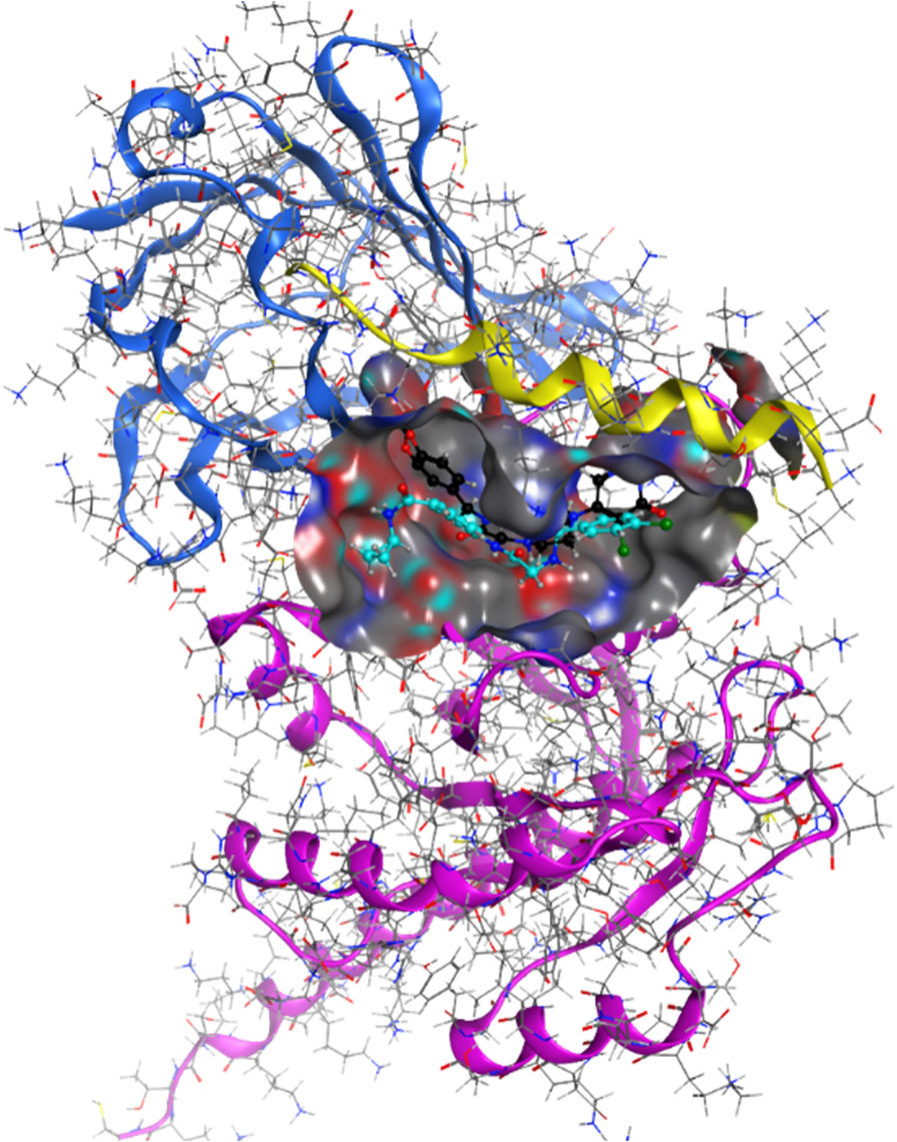
Interaction of ligands that stabilize the KRas4B-PDE6δ molecular complex in silico. The interprotein surface of interaction marked with gray color was used for the accomplishment of docking, being this the region with greater contact between KRas4B GTPase (pink and yellow) and PDE6δ (blue). The compounds D14 (black) and C22 (aqua) were identified in the same inter-protein interactions sites. (Molecular Operating Environment (MOE), 2014.09)

The primary and natural interaction between KRas4B and PDE6δ is through the farnesyl group attached to the C terminus of the former [22]. Our strategy does not consist in compete with the farnesyl binding site in PDE6δ to destabilize the complex, but on the contrary, to strengthen their binding through the design of a small drug-like ligand able to simultaneously bind to both molecules, in the interface of the complex, acting as a molecular staple. Binding of this type of ligand would reduce the dissociation rate of KRas4B and PDE6δ, and increase the affinity constant, thus affecting their functional role. To identify this type of compounds, we used virtual screening on the KRas4B-PDE6δ complex obtained from crystallographic data (PDB ID 5TAR25). The protein-protein interface area consists of 1900 Å^2^, with 12 interchain hydrogen bonds and 115 non-bonded contacts. We directed the molecular docking efforts to the groove formed around the interchain contact of the complex (Fig. 1). The surface of this groove was explored by MOE_SiteFinder where we identified several binding pockets composed of atoms of both proteins with a potential capacity of binding drug-like compounds.

### Virtual screening and docking interaction

**Fig. 2:**
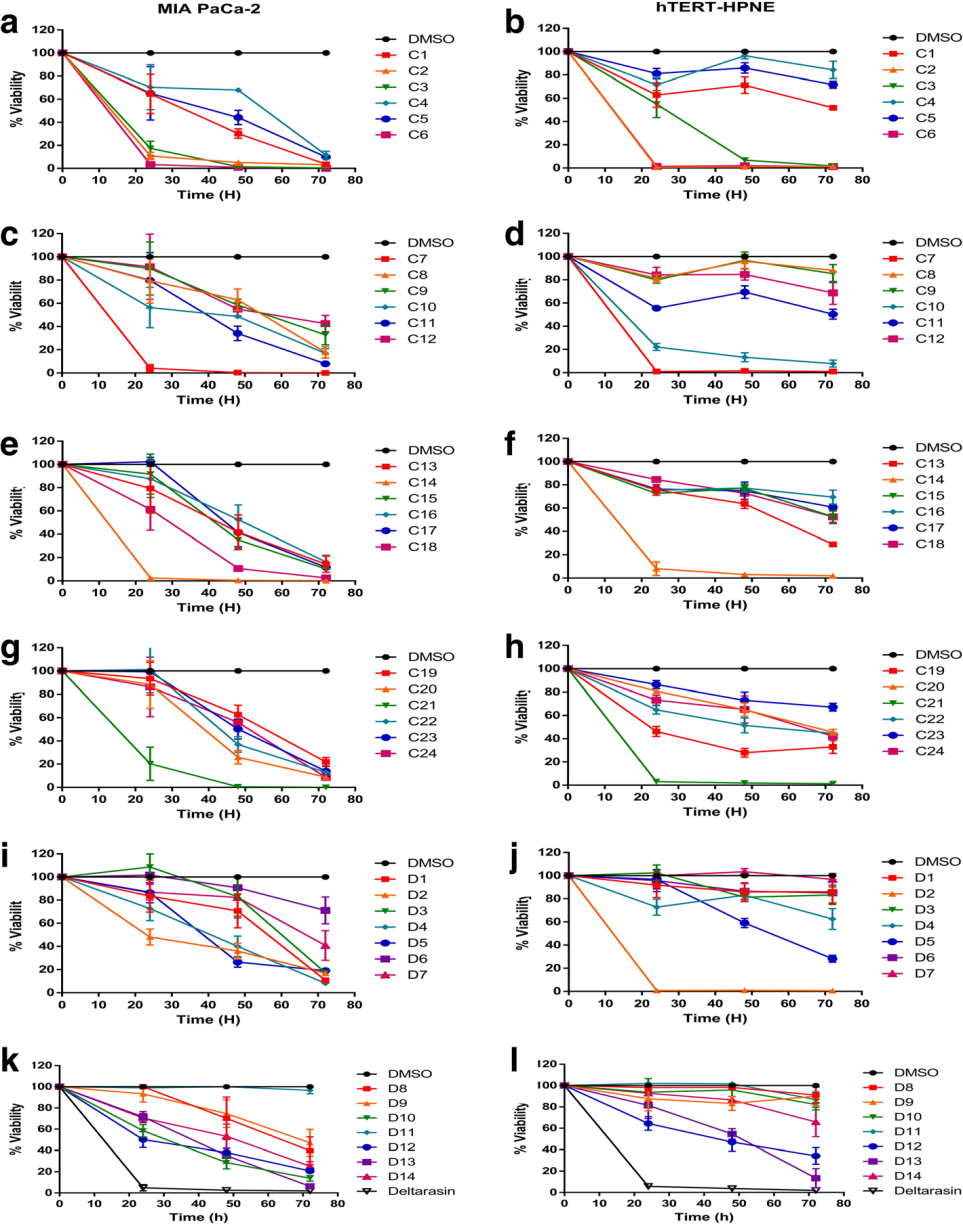
Identification and evaluation on the cellular viability of the compounds with greater interaction energy on the interprotein region compared with the effect of Deltarasin. Evaluation of 38 compounds at 200 μM, Deltarasin at 5 μM and DMSO as vehicle, on the MIA PaCa-2 (**a**, **c**, **e**, **g**, **i** and **k**) and hTERT-HPNE (**b**, **d**, **f**, **h**, **j** and **l**) cell lines

**Fig. 3:**
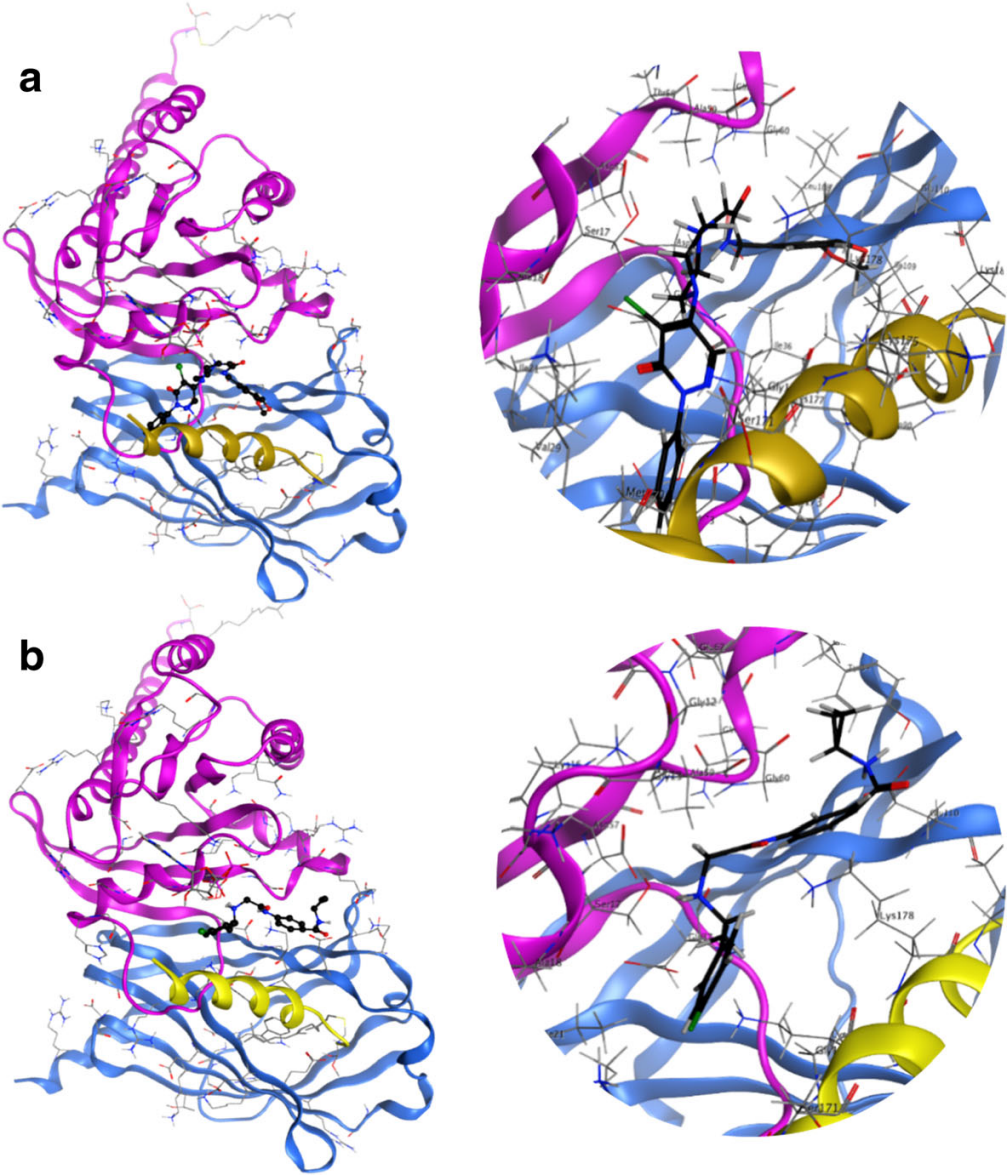
In silico interaction of compounds D14 and C22 at the interface of the protein complex KRas4B-PDE6δ. **a** Binding poses of lead molecule D14. **b** Compound C22 interacting with the complex. Both compounds establish different contacts at the inter-protein region of the molecular complex. (Molecular Operating Environment (MOE), 2014.09*)*

The database of conformers prepared from ENAMINE diversity dataset (50,240 compounds) was used for docking studies against the interfacial groove of the KRas4B-PDE6δ complex. The docking results were sorted and analyzed by binding score, frequency of obtaining similar poses, type of interactions, and presence of ligand contacts with both KRas4B and PDE6δ proteins. We gathered a group of 38 compounds fulfilling all requirements, 35 of them with optimal docking scores between − 13.4 and − 16.6 (Additional file 1: Table S1). All 38 compounds were acquired from ENAMINE for in vitro assays (Fig. 2). Also, we selected two of the compounds for further computational and binding-energy characterization by molecular dynamics. The choice of these compounds was based on a compromise of the different requirements used in the original selection, followed by visual inspection of the docked ligands on their interfacial binding-sites of the KRas4B-PDE6δ complex to form specific contacts between both proteins. We selected D14 as the compound with the highest molecular weight in the set, and thus with more atoms to be in potential contact to the protein complex. Also, we chose C22 as a representative compound of many of the poses obtained from docking with the whole set of compounds (Figs. 1 and 3). D14 showed a coupling of − 15.2. This molecule can have eleven hydrogen bonds with KRas4B: Gly15, Ser17, Glu31, Glu37, Asp38, Asp57, Gly60, Met169, Lys177 and Lys179, while PDE6δ has two hydrogen bonds: Leu105 and Glu107; presents an ionic bond with Asp57 of KRas4B; and six pi bonds with KRas4B: Ser17, Val29, Tyr32, Ile36, Gly60 and Ser181 and a pi bond with Glu107 of PDE6δ. Similarly, C22 has a score of − 13.9. This molecule has six hydrogen bonds with KRas4B: Ser17, Asp30, Met169, Asp172, Lys177 and Lys178, while PDE6δ has a hydrogen bond with Glu107; three pi bonds with KRas4B: Val29, Gly173 and Lys178 and a pi bond with Glu107 of PDE6δ (Table 1 and Fig. 3). These two compounds share a hydrogen bond with PDE6δ in Glu107, three hydrogen bonds with KRas4B in Ser17, Met169 and lys177 and a pi bond with KRas4B in Val29 (Table 1 and Fig. 3). The piperazine and acetamide groups of D14 are essential to interact with the molecular complex. In the case of the C22 compound, benzamide and the amino group are important for the interaction with the complex. These regions generated strong bonds, according to the results obtained in silico.

**Table 1:** Two of the possible candidates to stabilize the KRas4B-PDE6δ complex. Results obtained from the virtual selection analysis in heterodimeric crystallographic complex

Ligand	D14	C22
Structure		
IUPAQ	N-[(2H-1,3-benzodioxol-5-yl)methyl]-2-[4-(5-chloro-6-oxo-1-phenyl-1,6-dihydropyridazin-4-yl)piperazin-1-yl]acetamide	3-(2-{[1-(4-chlorophenyl)ethyl]amino}acetamido)-N-cyclopropylbenzamide
Formula/Molecular weight (g/mol)	C_24_H_24_ClN_5_O_4_/481.9	C_20_H_22_ClN_3_O_2_/371.9
Docking score (kcal/mol)	−15,2	−13,8
Hydrogen bond (kcal/mol) PDE6δ::Compound::	LEU 105 (−0,42) GLU 107 (− 0,3)	GLU 107 (− 2,63)
Hydrogen bond (kcal/mol) KRas4B::Compound	GLY 15 (− 0,285) SER 17 (− 0,25) GLU 31 (− 0,3) GLU 37 (− 0,8) ASP 38 (− 1,75) ASP 57 (− 7,1) GLY 60 (− 1,66) MET 169 (− 0,275) LYS 177 (− 0,5) LYS 179 (− 0,2)	SER 17 (0,1) ASP 30 (− 0,1) MET 169 (− 0,6) ASP 172 (− 0,42) LYS 177(− 0,2) LYS 178 (− 2,3)
Ionic bond (Å)	ASP 57 (− 9,9)	
Pi bond (kcal/mol) PDE6δ:: Compound	TRP 88 (−0,25)	GLU 107 (− 0,6)
KRas4B::Compound	SER 17 (−0,27) VAL 29 (− 0,2) TYR 32 (− 0,35) ILE 36 (− 0,3) GLY 60 (− 0,45) SER 181 (− 0,2)	VAL 29 (−0,4) GLY 173 (− 0,55) LYS 178 (−,46)

### Molecular dynamics and free energy calculations

Since all atoms of the receptor complex remained fixed during the docking procedure, we performed all-atom molecular dynamics (MD) simulations to introduce the effect of ligands on the heterodimeric complex and estimated the stability of the binding of both C22 and D14 on the native KRas4B-PDE6δ complex and the complex containing the G12C mutation in KRas4B. The results from these simulations allowed us to calculate the binding free-energy of each ligand to the complex, as well as the effect on KRas4B-PDE6δ binding in the presence and absence of the ligands (Table 2). Deviations from the starting structure (measured as RMSD) and increases in the molecular size (measured by the radius of gyration, RG) were calculated on snapshots from the MD to determine the equilibrium conditions in the simulations. This step is necessary to perform confident structural and energetic analyses of the protein-protein complex and the ligand-complex systems. The RMSD analysis demonstrated that the native and mutant KRas4B-PDE6δ, KRas4B-PDE6δ-D14 and KRas4B-PDE6δ-C22 systems reached equilibrated RMSD fluctuations during the first 10 to 50 ns with RMSD values that oscillated between 2.6 to 4.2 Å (Additional file 2: Table S2). RG analysis showed that the systems mentioned above reached equilibrated radius of gyration fluctuations after 20 to 50 ns with RG values that fluctuated between 22.2 to 22.8 Å (Additional file 2: Table S2). Correspondingly, RMSD analysis of the PDE6δ-ligand systems showed that systems reached an equilibrium fluctuation at 10 and 50 ns for PDE6δ-C22 and PDE6δ-D14, with average RMSD values of 1.9 ± 0.3 and 1.3 ± 0.2, respectively. RG analysis of the PDE6δ-C22 and PDE6δ-D14 systems demonstrated that they reached equilibrated RG oscillations at 50 and 20 ns, respectively, with average RG values of 16.4 ± 0.1 and 16.2 ± 0.1, respectively (Additional file 2: Table S2). RMSD and RG analyses of the protein-protein and protein-ligand systems showed that despite the differences in simulation time required to reach stable RMSD and RG values, all the systems finally reached equilibrium. Therefore, based on this structural performance, subsequent clustering analysis and binding free energy calculations were performed while excluding the first 50 ns from the 100-ns-long MD simulations (Table 2).

**Table 2:** Binding free energy components of protein-protein and protein-ligand complexes (in kcal/mol units)

System	ΔE_vdw_	ΔE_ele_	ΔG_ele,sol_	ΔG_npol,sol_	ΔE_non-polar_	ΔE_polar_	ΔG_bind_
Protein-protein Free and bound wild type KRas4B-PDE6δ complex							
KRas4B-PDE6δ	− 123.08 (0.35)	− 1546.94 (4.40)	1608.17 (4.25)	−17.78 (0.04)	− 140.86	61.23	−79.63 (0.43)
KRas4B-PDE6δ-D14	− 124.99 (0.44)	− 1127.75 (3.74)	1187.01 (3.50)	− 18.26 (0.05)	− 143.25	59.26	−83.99 (0.42)
KRas4B-PDE6δ-C22	− 142.17 (0.40)	− 1113.87 (4.30)	1185.59 (4.11)	−20.62 (0.03)	− 162.79	71.72	−91.07 (0.38)
Free and bound mutated KRas4BG12C-PDE6δ							
KRas4BG12C-PDE6δ	−127.74 (0.51)	− 1507.30 (5.58)	1550.55 (5.31)	−18.85 (0.06)	− 146.59	43.25	− 103.34 (0.77)
KRas4BG12C-PDE6δ-D14	−124.12 (0.51)	− 1083.80 (6.00)	1142.97 (5.68)	−18.00 (0.05)	−142.12	59.17	−82.95 (0.68)
KRas4BG12C-PDE6δ-C22	− 126.67 (0.39)	− 1550.02 (3.88)	1587.85 (3.75)	−18.86 (0.05)	− 145.53	37.83	−107.70 (0.50)
Protein-ligand							
PDE6δ-D14	−47.22 (0.15)	− 120.27 (0.95)	134.17 (0.86)	− 6.40 (0.02)	−53.62	13.90	−39.72 (0.20)
PDE6δ-C22	−41.76 (0.15)	−173.27 (0.47)	163.16 (0.38)	−6.54 (0.01)	−48.30	−10.11	− 58.41 (0.19)

Binding free energies and individual energy terms of complexes starting from docked conformations (kcal/mol). The polar (ΔEpolar = ΔEele + ΔGele,sol) and non-polar (ΔEnon-polar = ΔEvwd + ΔGnpol,sol) contributions are shown. All the energies are averaged over 500 snapshots at time intervals of 100 ps from the last 50 ns-long MD simulations and are in kcal/mol (± standard error of the mean)

### D14 and C22 compounds decrease cellular viability of pancreatic cancer cells

**Fig. 4:**
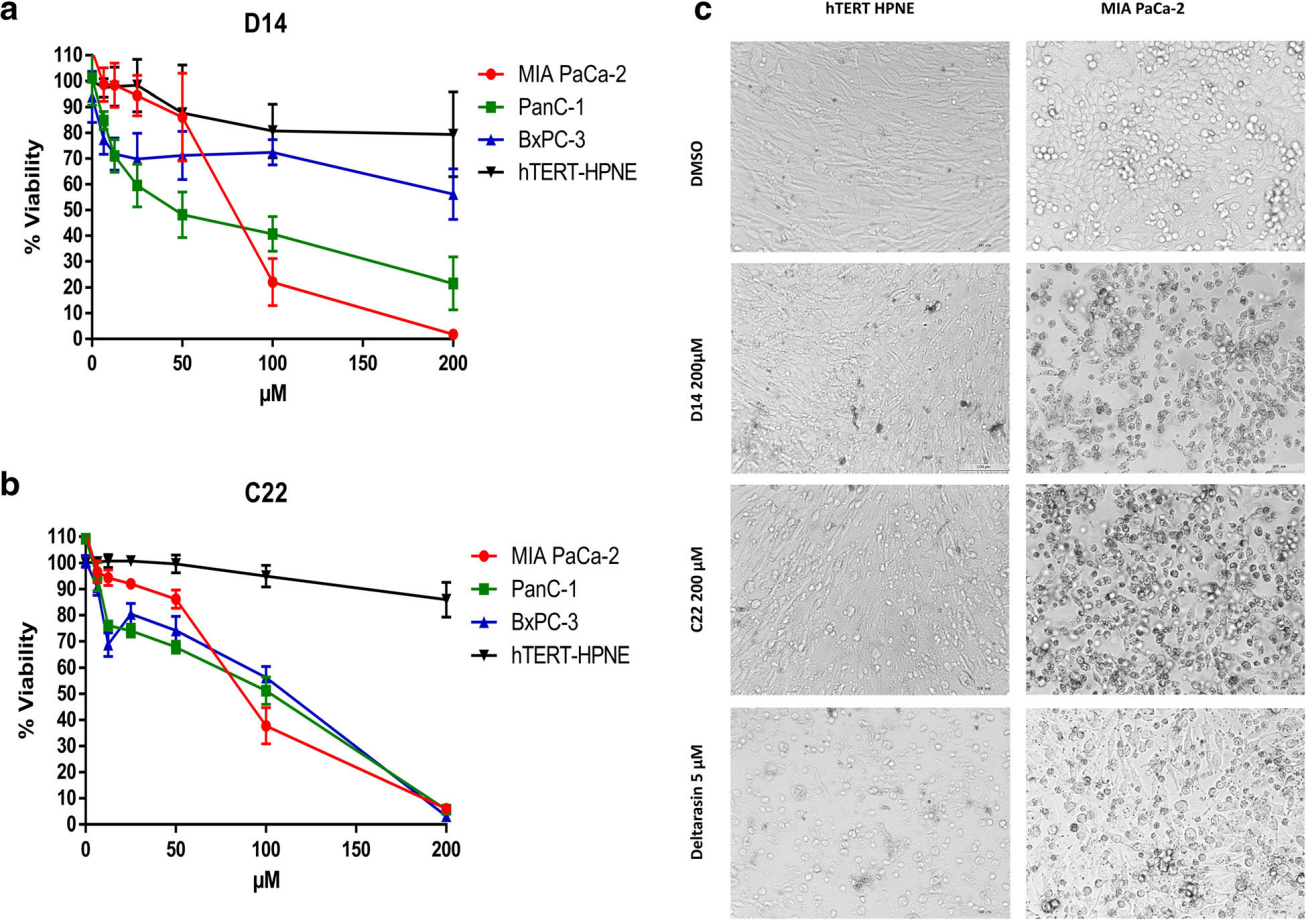
Compounds D14 and C22 decrease the cellular viability of KRas4B-dependent pancreatic cancer cells. **a** and **b** Effect of compounds D14 and C22 at various concentrations (0, 6.25, 12.5, 25, 50, 100 and 200 μM) for 72 h on MIA PaCa-2, BxPC-3 and PanC-1 pancreatic cancer cells and on hTERT-HPNE normal pancreatic cells. The results show the viability inhibition of pancreatic cancer cell induced by the treatments. **c** Morphological visualization of the MIA PaCa-2 and hTERT-HPNE cell lines treated at 200 μM of compounds D14 and C22

A cell viability assay was performed to determine whether compounds D14 and C22 have effects on cell viability or show cytotoxic effects. These compounds were tested on the hTERT-HPNE and MIA PaCa-2 cell lines at 200 μM, using Deltarasin (a PDE6δ inhibitor) as a positive control at 5 μM. After 72 h of incubation, we observed by microscopic analysis of the treated cell lines that these compounds have relatively high activity on the MIA PaCa-2 cell line without affecting the cellular hTERT-HPNE in terms of growth, morphology, proliferation, and the amount of live cells up to 72 h (Fig. 4c). D14 caused MIA PaCa-2 cells to peel off the dish surface, and their morphology did not change into a spherical shape. In the hTERT-HPNE cell line, cell detachment did not occur, and no drastic change in cell morphology was observed compared to DMSO control treatments. C22 caused similar damage to D14 in MIA PaCa-2 cells (Fig. 4c). However, the hTERT-HPNE cell line showed cell detachment and changes in morphology (Fig. 4c). Deltarasin affected the MIA PaCa-2 cell line, presenting round cells and less cell detachment than compounds D14 and C22. In the hTERT-HPNE cell line treated with Deltarasin, there was more cell detachment and spherical morphology than in the cells receiving other treatments, possible due to the inspecificity of Deltarasin. Therefore, it is possible that other proteins are being affected through a yet unknown molecular mechanism by Deltarasin. This effect was also verified by a cell viability assay in MIA PaCa-2 and hTERT-HPNE cells in the presence of different concentrations of D14 and C22 (6.25–200 μM) and DMSO treatments were followed for 5 days and read every 24 h. A dose-response effect was observed (Fig. 4a) with each treatment, and both compounds affected significantly the cell visibility of pancreatic cancer cell line MIA PaCa-2 more than the noncancerous pancreatic cell line hTERT-HPNE (Fig. 4a and b). We also evaluated the effects of both D14 and C22 compounds on cellular viability of BxPC-3 and PanC-1 pancreatic cancer cells lines, and found that both compounds affected viability of these pancreatic cancer cells. The IC_50_ values of compounds D14 and C22 on hTERT-HPNE cell line were 431.1 μM and 649.9 μM respectively, while IC_50_ to MIA PaCa-2, were 99.33 μM and 137.5 μM; to BxPC-3 were 252.85 μM and 97.88 μM; and PanC-1 cell lines were 105 μM and 108.5 μM. These results suggest that these compounds specifically affects cell viability of pancreatic cancer cell lines.

### D14 and C22 compounds induce apoptosis in cancer pancreatic cells lines

One of the objectives proposed in the present work was to determine the type of cell death produced by D14 and C22 compounds in the hTERT-HPNE and MIA PaCa-2 cells lines. To this end, we used two different experimental strategies, one of them was using the Apoptosis/Necrosis Detection kit analyzed by flow cytometry which allows to detect the double labeling of apoxin-V and 7-Aminoactinomycin D (7-AAD). The second was the Human Apoptosis Array kit to determine the impact of D14 and C22 compounds on phosphorylation of different elements associated with the signaling pathways of apoptosis.

**Fig. 5:**
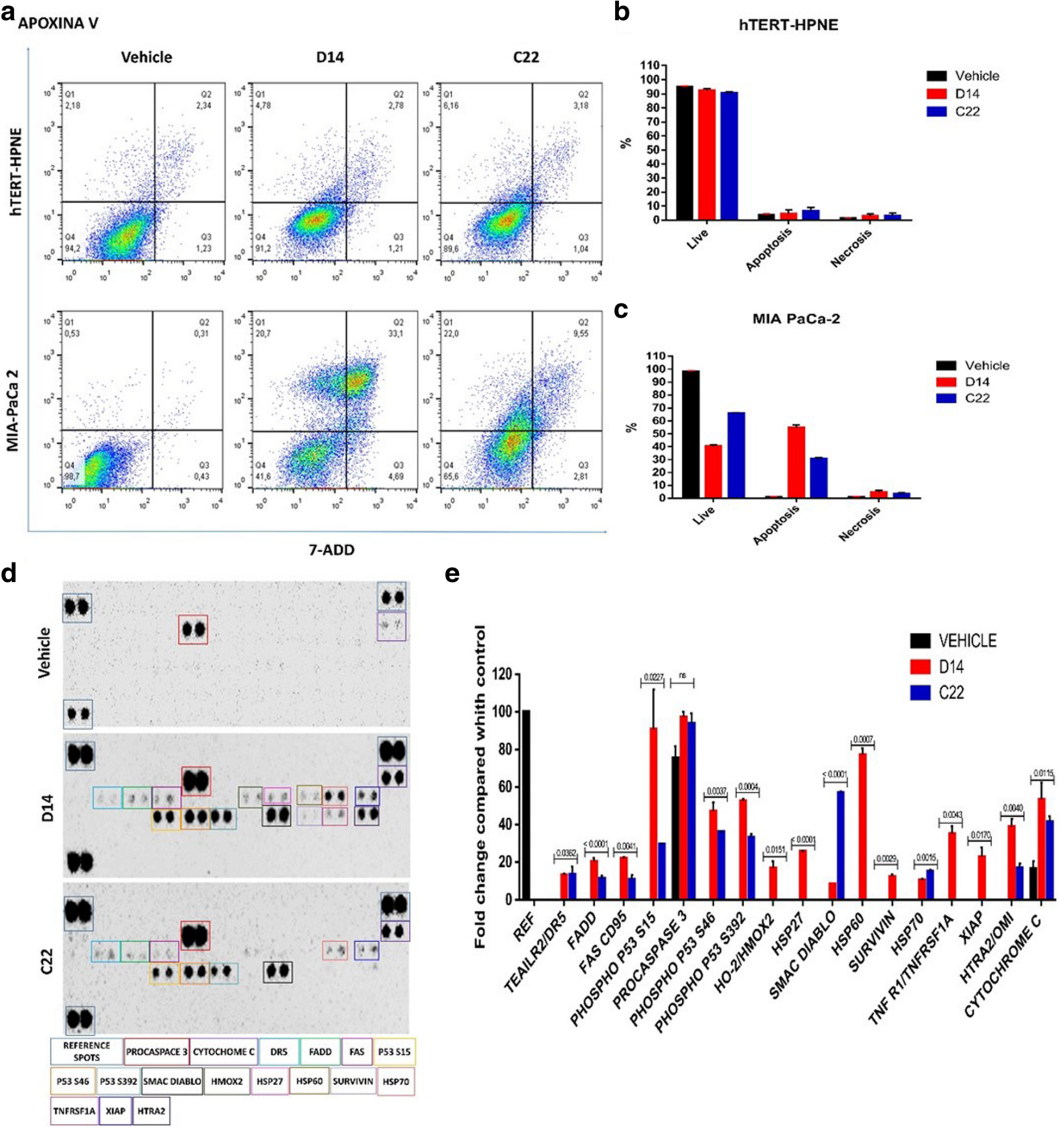
Compounds D14 and C22 induce apoptosis in the ondependent cell line of KRas4B. **a**, **b** and **c** Cell death of MIA PaCa-2 and hTERT-HPNE cells was determined by apoxin V / 7-AAD / CytoCalcein Violet and analyzed by flow cytometry. **d** Compounds D14 and C22 induced apoptosis by being reflected in the presence of the phosphorylation of P53, Cytochrome-c, procaspase 3, and Smac/Diablo on the MIA PaCa-2 cell line of KRas4B-deoendent. The phosphorylation of these proteins was significantly compared with the control. The total protein extract (300 μg) was used for the apootosis kit. The dots of the matrix were visualized according to the manufacturer’s instructions. The intensity of each point was measured as described in “Material and methods”. The upper panel provides matrix analysis of MIA PaCa-2 without compounds (control); the central panel shows the matrix analysis of the MIA PaCa-2 cell line treated with compound D14; the lower panel shows the matrix analysis of the MIA PaCa-2 cell line treated with the C22 compulsion. **e** Normalized quantification graph shows the relative change of the phosphorylation differences with respect to the control

Flow cytometry analysis showed that compound D14 promoted cell death to 53.8% by apoptosis and to only 4.69% by necrosis; and that compound C22 promoted cell death to 31.55% by apoptosis and to 2.81% by necrosis. By contrast compounds D14 and C22 caused no significant cell death in the normal pancreatic hTERT-HPNE cells, with 91.2 and 89.8% viability detected in normal pancreatic cells treated with D14 and C22 compounds, respectively (Fig. 4a, b and c). Moreover, D14 and C22 compounds promoted an increase in phosphorylation of the p53, S15, S46, and S392 residues of procaspase 3, Smac/Diablo and cytochrome-c (Fig. 5d and e). These results indicate that D14 and C22 compounds have the property to induce activation of proapoptotic proteins [23]. However, only D14 compound promoted phosphorylation of XIAP, which has been reported as inhibitor of apoptosis [24]. With this information, we consider that the C22 compound is better than D14, to promote specific cell death on pancreatic cancer cells.

### D14 and C22 compounds decrease Ras activity and inhibit AKT and ERK phosphorylation in pancreatic cancer cells

**Fig. 6:**
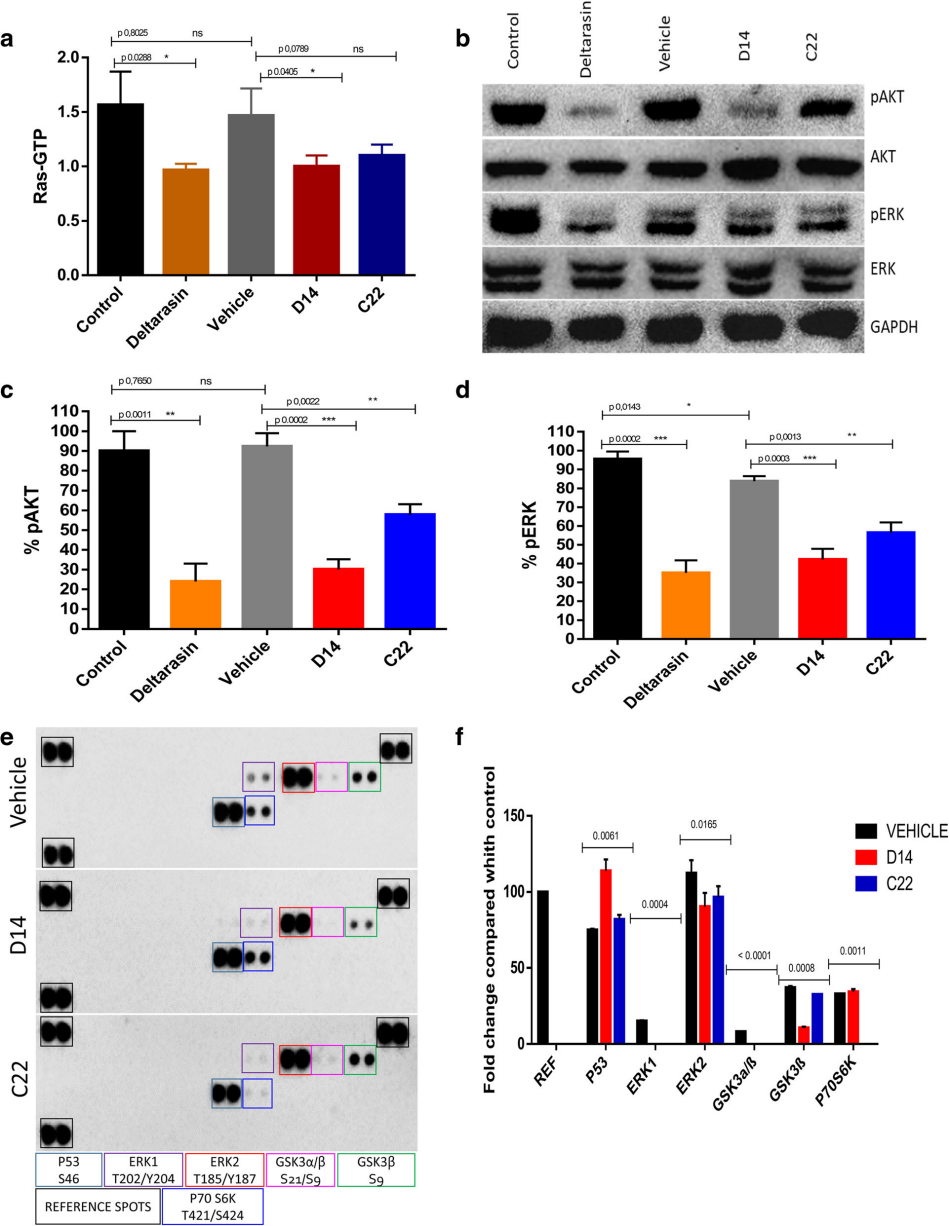
Compounds D14 and C22 decrease the activation of ras in ondependent cells of KRas4B and the phosphorylation of AKT and ERK promoting the increased phosphorylation of p53. **a** Ras activation (Ras-GTP) decreases in the MIA PaCa-2 cell line treated with the compounds D14 (99.33 μM), C22 (137.5 μM) and Deltarasin at 5 μM for 3 h. **b** Representative immunoblot of whole protein extracts from MIA PaCa-2 treated with D14, C22 and deltarasin for 3 h, detected phosphorylation inhibition of AKT and ERK using GAPDH with load control. **c** and **d** The quantitative results of the immunoblot of 3 independent studies are shown in graphs. **e** D14 and C22 compounds induced inhibition of the phosphorylation of ERK1 (T202/Y204), ERK2 (T185/Y187) in human MIA PaCa-2 cancer cell and promoted an increased in the P53 phosphorylation (**e** and **f**). The phosphorylation of those proteins were significantly inhibited compared with the control. Protein extract (300 μg) were used for human phospho-MAPK array kit. Array spots were visualized in accordance with the manufactures’s instructions. The intensity of each spot was measured as described in “.Material and Methods”. The upper panel gives the array analysis from MIA PaCa-2 without compounds (control); the middle panel shows the array analysis of MIA PaCa-2 cell line treated with D14 compound; the lower panel shows the array analysis of MIA PaCa-2 cell line treated with the C22 compoud. **e** Normalized quantitation graph that shows the relative fold change of phosphorylation differences upont for control

It has been reported that molecules downstream of KRas4B such as pAKT and pERK in pancreatic cancer cells are related to the signaling pathways involved in survival and cell differentiation. Therefore, we decided to analyze and explore the activity of compounds D14 and C22 on these signaling pathways, as well as on KRas4B, and to compare this activity to that of Deltarasin in the MIA PaCa-2 cell line. D14 and C22 and Deltarasin compounds showed that total Ras activation in the MIA PaCa-2 cell line was reduced by approximately 33% (Fig. 6a). Treatments with the D14 compound also showed a decrease of 70% in AKT phosphorylation and 51% in ERK phosphorylation (Fig. 6b and c), while compound C22 changed AKT phosphorylation by 60% and ERK phosphorylation by 36% (Fig. 6b and d). Deltarasin decreased AKT phosphorylation by approximately 75% and ERK phosphorylation by 54% (Fig. 6b, c and d). Also a phosphor-MAPK array kit was used to explore the possible mechanism of how D14 and C22 compounds induce cell viability inhibition. Detecting that three members of the MAPK pathway were involved in those effects such as pERK, pAKT, which showed a reduction in expression of phosphorylated forms of these enzymes, as well as an increase in the phosphorylated form of P53 (S46) protein (Fig. 6e and f). These data indicate that compounds D14 and C22 negatively impacted the activation of KRas signaling pathways in the MIA PaCa-2 cancer cell line.

### Compounds D14 and C22 inhibit tumor growth in a pancreatic cancer xenograft mouse model

**Fig. 7:**
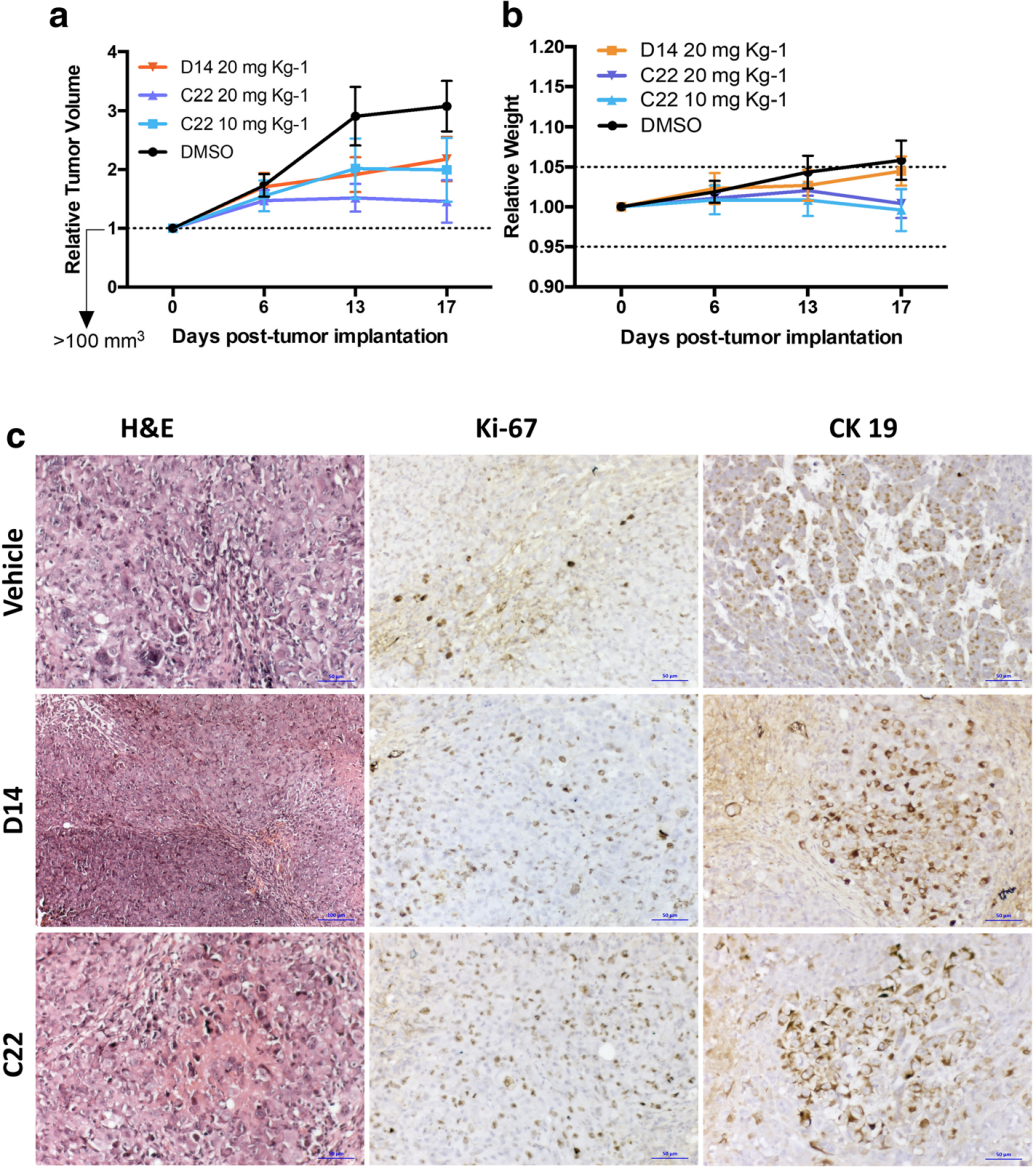
Compounds D14 and C22 decrease tumor growth in vivo. **a** Subcutaneous xenograft of cell MIA PaCa-2, the tumor volume was evaluated during the 15 days of treatment. **b** The Weight of the mice was measured throughout the treatment. The mice were treated with the vehicle and compounds D14 and C22 at the two indicated in the scatter plot. NuNu mice were treated with vehicle (10% DMSO, 0.05% carboxy methyl cellulose and 0.02% Tween 80), D14 at 20 mg kg-1 or C22 at 10 mg kg-1 or 20 mg kg- 1 administered by intraperitoneal injection every two days (*n* = 10 for DMSO, *n* = 7 for D14 at 10 mg kg-1, *n* = 5 for C22 at 10 mg kg-1 and n = 10 for C22 at 20 mg kg-1). Changes in tumor volume are given in relation to the initial volume before treatment (the dotted line indicates the initial size of the tumor). **c** Hematoxylin-eosin and immunohistochemistry of the tumor sections. The 2 μm sections of the tumors were stained with hematoxylin-eosin (H & E) and analyzed by immunohistochemistry with antibodies directed against cytokeratin 19 (CK19) and Ki-67

To evaluate the antitumor activity of compounds D14 and C22, Nu/Nu mice were inoculated subcutaneously with the pancreatic cancer cell line MIA PaCa-2, and tumor growth was monitored. The different treatments were administered intraperitoneally (i.p.) three times per week with a total of 7 injections in each mouse. Different doses (10 and 20 mg kg^− 1^) were tested (Fig. 7a). The results showed a tumor reduction of 88.6 and 65.9% in mice treated with compounds C22 and D14, respectively. The highest effect on tumor size decrease was observed at a dose of 20 mg kg^− 1^ of compound C22 compared to that of DMSO (control), this effect significantly increased on the last day (Fig. 7a). Moreover, compared to the treated mice, no mouse treated only with vehicle showed a reduction in tumor size. The treatment with compounds D14 and C12 did not cause weight loss in mice, and those that were treated lost less than 5% of their total body weight (Fig. 7b). Deltarasin causes a 15% weight decrease in mice during the first two days of treatment (data not shown). The histopathological analysis of in vivo evaluation of D14 and C22 compounds (Additional file 3: Table S3) allowed to determine that these compounds reduced the presence of human pancreatic cancer cells by 60 and 70% respectively (Fig. 7c). All data suggest that compounds D14 and C22 are non-toxic and shown that the D14 and C22 compounds have antineoplasic properties specific against pancreatic cancer cells.

## Discussion

Pancreatic cancer is one of the most lethal cancers in the world, and it has been observed that the expression of mutated KRas4B is sufficient for the development and tumor growth in pancreatic cancer as well as for one-third of other types of cancers. Mutations at the 12th, 13th and 61st residues in the small GTPase KRas4B limit the function of its molecular negative regulator RasGAP, so the activity of the GTPase KRas4B, as well as the signaling pathways dependent on this GTPase, remains constitutively active. The possibility of identifying compounds that bind directly to KRas4B and block its function has been studied for more than three decades; however, the efforts made to find compounds that inhibit the activity of mutated KRas4B activity have not been successful, which is mainly due to the lack of small-molecule binding sites on its KRas4B molecular target. Thus, in this work, the most important goal was to detect in silico compounds stabilizing the molecular complex KRas4B-PDE6δ, as well as to evaluate the antineoplastic properties of these compounds on the pancreatic cancer MIA PaCa-2 cell line, for the first time. For this purpose, we detected 38 compounds. In the present work, we only reported the in vitro and in vivo evaluation of compounds D14 and C22. However, it is important to point out that we have deepened the study on other compounds identified in the present work at the preclinical stage, and the results have been consistent and promising regarding specific antineoplasic properties, since these compounds not only affected the pancreatic cancer cells but also showed KRas4B dependent activity on colon cancer cells. Regarding the in silico analysis of compounds D14 and C22, it was possible to determine the binding free energy (ΔG_bind_) for the protein-protein and receptor-ligand complexes, and the binding of all the systems were found to be thermodynamically favorable. Table 2 illustrates that the primary energetic contribution to ΔG_bind_ for protein-protein and protein-ligand systems was guided by nonpolar contributions (ΔE_non-polar_). In contrast, the polar contributions (ΔE_polar_) showed unfavorable energy influences on all the protein-protein complexes but not the protein-ligand contacts. Comparison among the different protein-protein systems demonstrates that in our computer simulations, C22 is more efficient in promoting the affinity between KRas4B and PDE6δ than D14. Mutated KRas4B increased its affinity to PDE6δ with respect to wild-type, the association of D14 to mutated KRas4B-PDEδ complex contributed to decrease the affinity of the mutated KRas4B-PDEδ complex, whereas an increased in the affinity was observed when C22 was bound to the mutated heterodimer. Overall, comparison of the results for the wild-type and mutated systems highlight that the two compounds are efficient in increasing the protein-protein association of wild-type KRas4B-PDE6δ complex, whereas only C22 is able to increase the protein-protein association in wild-type and mutated KRas4B-PDE6δ complex. Although the in silico results indicate that these compounds may bind to the KRas4B-PDE6δ complex, increasing its interaction and thus affecting its function, we also demonstrate these effects experimentally. The results show that compounds D14 and C22 decrease total Ras activity and directly impact AKT and ERK. The experimental data suggest that compounds D14 and C22 impact cellular processes related to survival, cell cycle, protein synthesis and cell growth. The inhibition of the AKT signaling pathway could explain the induction of apoptosis by compounds D14 and C22 in the MIA PaCa-2 cell line, whereas the inhibition of ERK by compound D14 and C22 would influence transcriptional regulation of signaling pathways and cell cycle regulation. Despite the differential effects of compounds D14 and C22, these compounds directly impact the phosphorylation status of at least two signaling pathways that have been reported to be vital in pancreatic cancer cells and whose constitutive activation is associated with a bad prognosis in patients with pancreatic cancer [25]. On the other hand, the D14 and C22 compounds promoted a differential activation of elements related with apoptosis signaling pathways in the pancreatic cancer cells such as p53, procaspase 3, Smac/Diablo and cytochrome-c. However, D14 compound promoted an increase in the phosphorylation of XIAP protein, which has been reported to be an inhibitor of apoptosis [24]. Thus, we consider that C22 compound could be better than D14. The antineoplasic activity of D14 and C22 compounds detected in vitro also revealed its inhibitory activity against tumor growth in a xenograft murine model. In these trials, it is important to note that the mice did not exhibit weight loss during the treatment, suggesting that the compound does not have a toxic effect in animals. However, in the near future, pharmacokinetics and biodistribution studies on compounds D14 and C22 in mice will be necessary in order to evaluate the therapeutic use of these compounds. It will also be important to explore the combined effect of compounds D14 and C22 and determine whether there is a synergistic antineoplastic effect between the two compounds on pancreatic cancer cells. It is also important to note that we have selected and deepened our knowledge of the antineoplasic properties of other organic compounds presented in this work, which have shown specific and improved antineoplasic potency against pancreatic cancer cells, as well as analogs of these compounds. All these data show the great potential of the antineoplasic properties of the compounds evaluated in the present work.

## Conclusions

For the first time, we report two small molecules that stabilize the KRas4B-PDE6δ molecular complex. The antineoplastic evaluation of these compounds showed that they affected Ras activation pathways and tumor growth in xenografted mice. The antineoplastic activity was specifically against pancreatic cancer cells, as normal pancreatic cells were not affected. Compounds D14 and C22 present a new pharmacological alternative for suppressing Ras signaling in pancreatic cancer cells and for developing novel drugs against KRas4B-dependent pancreatic cancer. However, additional experiments are needed to allow us to unravel the specific mechanisms of action of the compounds reported in this work.

## Additional files

Additional file 1: **Table S1.** Potential candidates to stabilize the KRas4B-PDE6δ complex. Results obtained from the virtual screening analysis. Frequency stands for the number of times a similar pose was obtained from different starting conditions during our docking procedure. (DOC 477 kb)

Additional file 2: **Table S2.** The time-dependence of RMSD and RG by D14 and C22 compounds that target wild-type and mutated KRas4B-PDE6δ molecular complexes. (DOC 35 kb)

Additional file 3: **Table S3.** Summary of histopathological analysis and in vivo evaluation by the D14 and C22 compounds on tumor growth in nude mice. (DOC 30 kb)

## References

[1] Siegel R, Ma J, Zou Z, Jemal A (2014). Cancer. Statistics 2014. CA Cancer J Clin.

[2] Moskaluk CA, Hruban RH, Kern SE (1997). p16 and K-ras gene mutations in the intraductal precursors of human pancreatic adenocarcinoma. Cancer Res.

[3] Collins MA, Bednar F, Zhang Y, Brisset JC, Galban S, Galban CJ, Rakshit S, Flannagan KS, Adsay NV, Pasca di Magliano M (2012). Oncogenic Kras is required for both the initiation and maintenance of pancreatic cancer in mice. J Clin Invest.

[4] Collins MA, Brisset JC, Zhang Y, Bednar F, Pierre J, Heist KA, Galban CJ, Galban S, di Magliano MP (2012). Metastatic pancreatic cancer is dependent on oncogenic Kras in mice. PLoS One.

[5] Chandra A, Grecco HE, Pisupati V, Perera D, Cassidy L, Skoulidis F, Ismail SA, Hedberg C, Hanzal-Bayer M, Venkitaraman AR, Wittinghofer A, Bastiaens PI (2012). The GDI-like solubilizing factor PDEδ sustains the spatial organization and signalling of Ras family proteins. Nat Cell Biol.

[6] Zimmermann G, Papke B, Ismail S, Vartak N, Chandra A, Hoffmann M, Hahn SA, Triola G, Wittinghofer A, Bastiaens PI, Waldmann H (2013). Small molecule inhibition of the KRAS–PDEδ interaction impairs oncogenic KRAS signalling. Nature.

[7] Spiegel J, Cromm PM, Zimmermann G, Grossmann TN, Waldmann H (2014). Small-molecule modulation of Ras signaling. Nat Chem Biol.

[8] Papke B, Murarka S, Vogel HA, Martín-Gago P, Kovacevic M, Truxius DC, Fansa EK, Ismail S, Zimmermann G, Heinelt K, Schultz-Fademrecht C, Al Saabi A, Baumann M, Nussbaumer P, Wittinghofer A, Waldmann H, Bastiaens PI (2016). Identification of pyrazolopyridazinones as PDEδ inhibitors. Nat Commun.

[9] Vilar S, Cozza G, Moro S (2008). Medicinal chemistry and the molecular operating environment (MOE): application of QSAR and molecular docking to drug discovery. Curr Top Med Chem.

[10] Liang J, Edelsbrunner H, Woodward C (1998). Anatomy of protein pockets and cavities: measurement of binding site geometry and implications for ligand design. Protein Sci.

[11] Case DA, Cheatham TE, Darden T, Gohlke H, Luo R, Merz KM, Onufriev A, Simmerling C, Wang B, Woods RJ (2005). The Amber biomolecular simulation programs. J Comput Chem.

[12] Duan Y, Wu C, Chowdhury S, Lee MC, Xiong G, Zhang W, Yang R, Cieplak P, Luo R, Lee T, Caldwell J, Wang J, Kollman P (2003). A point-charge force field for molecular mechanics simulations of proteins based on condensed-phase quantum mechanical calculations. J Comput Chem.

[13] Wang J, Wolf RM, Caldwell JW, Kollman PA, Case DA (2004). Development and testing of a general amber force field. J Comput Chem.

[14] Jorgensen W.L, J. Chandrasekhar, J, Madura, J.D. Comparison of simple potential functions for simulating liquid water, J Chem Phys 1983;79:926–935.

[15] W.F. Van Gunsteren W.F, Berendsen H.J.C. Algorithms for macromolecular dynamics and constraint dynamics, Mol Phys 1977;34:1311–1327.

[16] Darden T, York D, Pedersen L (1993). Particle mesh Ewald: an N. log(N) method for Ewald sums in large systems. J Chem Phys.

[17] Berendsen HJC, Postma JPM, van Gunsteren WF, DiNola A, Haak JR (1984). Molecular-dynamics with coupling to an external bath. J Chem Phys.

[18] Miller B.R 3rd, McGee T.D.Jr, Swails J.M, Homeyer N, Gohlke H, Roitberg A.E. MMPBSA.Py: an efficient program for end-state free energy calculations. J Chem Theory Comput 2012;8:3314–3321. 10.1021/ct300418h 26605738

[19] Gohlke H, Case DA (2004). Converging free energy estimates: MM-PB(GB)SA studies on the protein-protein complex Ras-Raf. J Comput Chem.

[20] Kollman PA, Massova I, Reyes C, Kuhn B, Huo S, Chong L, Lee M, Lee T, Duan Y, Wang W, Donini O, Cieplak P, Srinivasan J, Case DA, Cheatham TE (2000). Calculating structures and free energies of complex molecules: combining molecular mechanics and continuum models. Acc Chem Res.

[21] Onufriev A, Bashford D, Case DA (2004). Exploring protein native states and large-scale conformational changes with a modified generalized born model. Proteins.

[22] Dharmaiah S, Bindu L, Tran TH, Gillette WK, Frank PH, Ghirlando R, Nissley DV, Esposito D, McCormick F, Stephen AG, Simanshu DK (2016). Structural basis of recognition of farnesylated and methylated KRAS4b by PDEδ. Proc Natl Acad Sci U S A.

[23] Cailleteau C, Liagre B, Beneytout JL (2009). A proteomic approach to the identification of molecular targets in subsequent apoptosis of HEL cells after diosgenin-induced megakaryocytic differentiation. J Cell Biochem.

[24] Li S, Sun J, Yang J, Zhang L, Wang L, Wang X, Guo Z (2013). XIAP expression is associated with pancreatic carcinoma outcome. Mol Clin Oncol.

[25] Chadha KS, Khoury T, Yu J, Black JD, Gibbs JF, Kuvshinoff BW, Tan D, Brattain MG, Javle MM (2006). Activated Akt and Erk expression and survival after surgery in pancreatic carcinoma. Ann Surg Oncol.

