# Supplementary material for: KRas4B-PDE6δ complex stabilization by small molecules obtained by virtual screening affects Ras signaling in pancreatic cancer
Source: BMC Cancer. 2018 Dec 29;18:1299. doi: 10.1186/s12885-018-5142-7 (PMC6310981; doi:10.1186/s12885-018-5142-7)
Supplement: Supplementary file 1 — Table S1. Potential candidates to stabilize the KRas4B-PDE6δ complex. Results obtained from the virtual screening analysis. Frequency stands for the number of times a similar pose was obtained from different starting conditions during our docking procedure. (DOC 477 kb) [file 12885_2018_5142_MOESM1_ESM.doc]

**Table S1.** **Potential candidates to stabilize the KRas4B-PDE6δ complex.** Results obtained from the virtual screening analysis. Frequency stands for the number of times a similar pose was obtained from different starting conditions during our docking procedure.

| **Ligand name**  **ENAMINE ID** | | **Structure** | **Molecular weigth**  **(g/mol)** | **Docking score** | **Frequency** | **CLogP** |
| --- | --- | --- | --- | --- | --- | --- |
| D1  Z52714899 | | *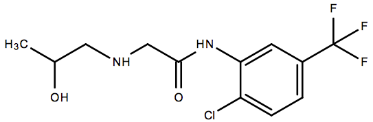*  *[2-[2-chloro-5-(trifluoromethyl)anilino-2-keto-ethyl]-[(2S)-2-hydroxypropyl]ammonium* | 310,7 | -13,1 | 17 | 3.268 |
| D2  Z56794607 | | 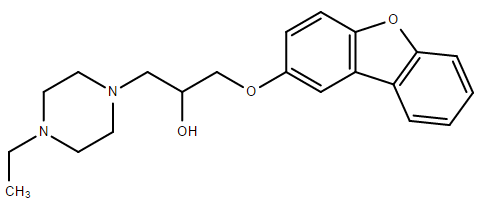  *(2S)-1-dibenzofuran-2-yloxy-3-(4-ethylpiperazin-1-yl)propan-2-ol* | 354,4 | -16,6 | 328 | 2.907 |
| D3  Z51185275 | | 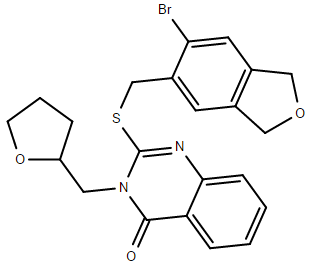  *2-[(6-bromobenzo[1,3]dioxol-5-yl)methylsulfanyl]-3-(tetrahydrofuran-2-ylmethyl)quinazolin-4-one* | 475,4 | -5,0 | 1 | 4.707 |
| D4  Z104377870 | | 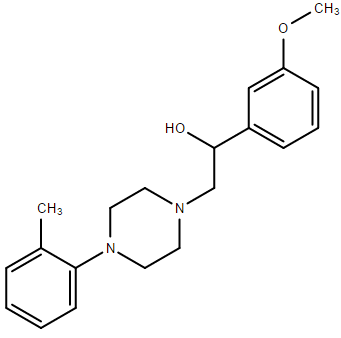  *(1R)-1-(3.methoxyphenyl)-2-[4-(o-tolyl)piperazin-1-yl]ethanol* | 326,4 | -15,5 | 11 | 3.581 |
| D5  Z90661758 | | 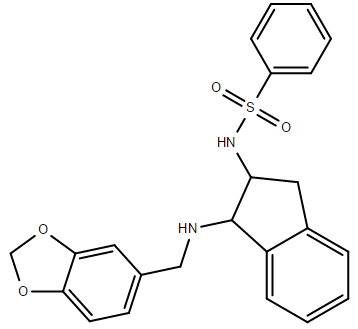  *N-[(1S,2R)-1-(1,3-benzodioxol-5-ylmethylamino)indian-2-yl]benzenesulfonamide* | 422,5 | -14,5 | 5 | 3.796 |
| D6  Z44508405 | | 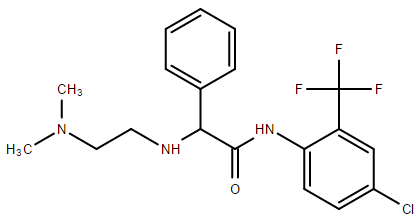  *(2S)-N-[4-chloro-2-(trifluoromethyl)phenyl]-2-(2-dimethylaminoethylamino)-2-phenyl-acetamide* | 399,8 | -13,8 | 192 | 3.298 |
| D7  Z118602326 | | 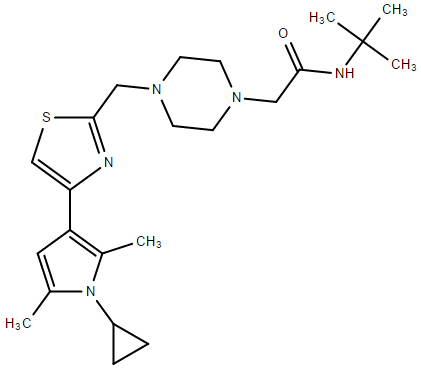  *N-tert-butyl-2-[4-[[4-1-cyclopropyl-2,5-dimethyl-pyrrol-3-yl)thiazol-2-yl]methyl]piperazin-1-yl]ace* | 429,6 | -14,5 | 55 | 3.735 |
| D8  Z56779938 | | 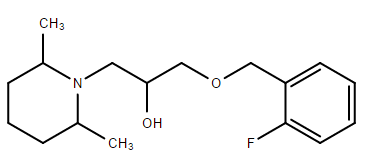  *1-(2,6-dimethyl-1-piperidinyl)-3-[(2-fluorophenyl)methoxy]propan-2-ol* | 295,4 | -13,8 | 8 | 3.533 |
| D9  Z149257580 | | 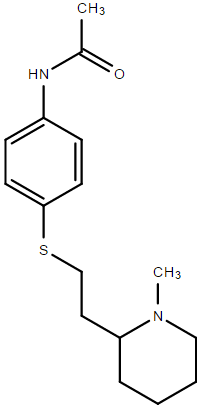  *N-[4-[2-(1-methyl-2-piperidyl)ethylsulfanyl]acetamide]* | 292.4 | -12,2 | 1 | 3.251 |
| D10  Z25944554 | | *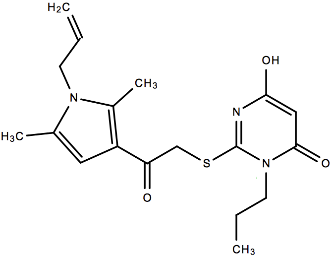*  *2-({2-[2,5-dimethyl-1-(prop-2-en-1-yl)pyrrol-3-yl]-2-oxoethyl}sulfanyl)-6-hydroxy-3-propylpyrimidin-4-one* | 361,5 | -15,6 | 373 | 3.405 |
| D11  Z27029312 | | 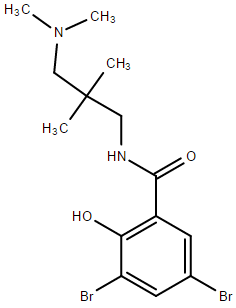  *[3-[(3, 5-dibromo- 2-hydroxy- benzoyl) amino]-2,2-dimethyl- propyl]- dimethyl- ammonium* | 408,1 | -13,8 | 20 | 4.638 |
| D12  Z31373014 | | 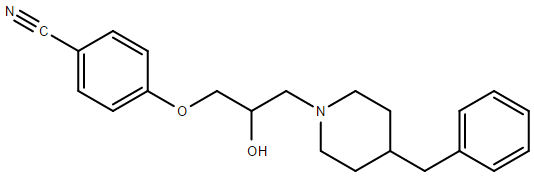  *4-[3-(4-benzyl-1-piperidyl)-2-hydroxy-propoxy]benzonitrile* | 350,5 | -13,9 | 15 | 4.233 |
| D13  Z105308834 | | 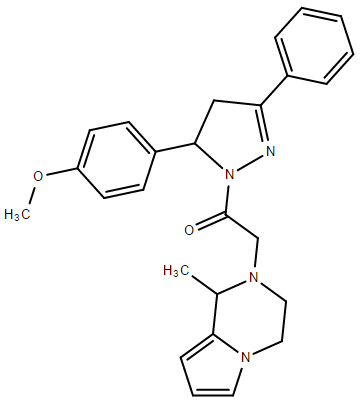  *1-[(3S)-3-(4.methoxyphenyl)-5-phenyl-3,4-dihydropyrazol-2-yl]-2-[(1R)-1-methyl-3,4-dihydro-1H-pyrrol* | 428,5 | -13,8 | 8 | 4.744 |
| D14  **Z46515334** | | 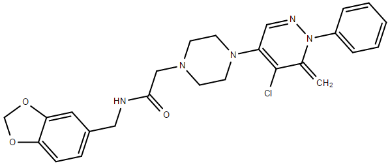  *N-(1,3-benzodioxol-5-ylmethyl)-2-[4-(5-chloro-6-oxo-1-phenyl-pyridazin-4-yl)piperazin-1-yl]acetamide* | 481,9 | -15,2 | 24 | 3.129 |
| C1  Z146241516 | | 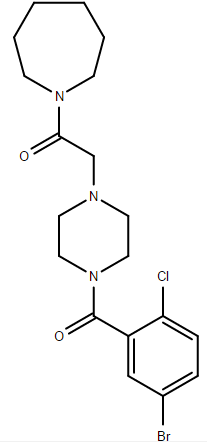  *1-(azepan-1-yl)-2—[4-(5-bromo-2-chloro-benzoyl)piperazin-1-yl]ethanone* | 442,8 | -13,6 | 43 | 4.119 |
| C2  Z90250319 | | 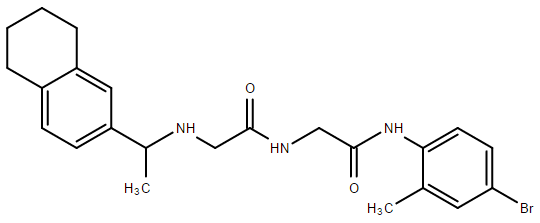  *N-(4-bromo-2-methyl-phenyl)-2-[[2-[[(1R)-1-tetralin-6-ylethyl]amino]acetyl]amino]acetamide* | 450,4 | -14,0 | 33 | 4.756 |
| C3  Z105370208 | | 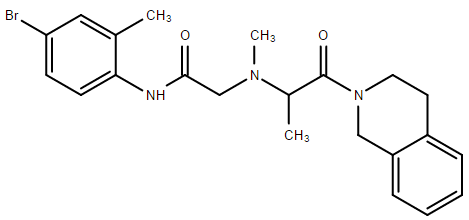  *N-(4-bromo-2-methyl-phenyl)-2-[[(1R)-2-(3,4-dihydro-1H-isoquinolin-2yl)-1-methyl-2-oxo-ethyl]-methy* | 444,4 | -13,9 | 22 | 3.805 |
| C4  Z607177054 | | 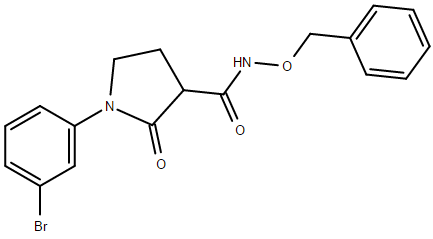  *(3R)-N-benzyloxy-1-(3-bromophenyl)-2-oxo-pyrrolidine-3-carboxamide* | 389,2 | -14,9 | 215 | 3.948 |
| C5  Z786305506 | | 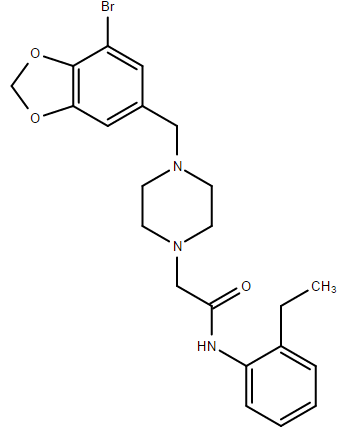  *2-4[-[7-bromo-1,3-benzodioxol-5-yl)methyl]piperazin-1-yl]-N-(2-ethylphenyl)acetamide* | 460,4 | -15,8 | 422 | 4.633 |
| C6  Z234893597 | | 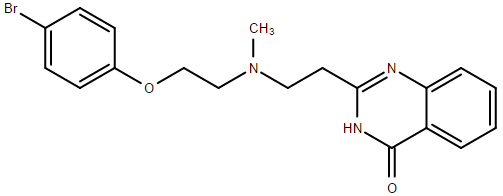  *2-[2-[2-(4-bromophenoxy)ethyl-methyl-amino]ethyl]-3H-quinazolin-4-one* | 402,3 | -13,5 | 32 | 3.423 |
| C7  Z31349966 | | 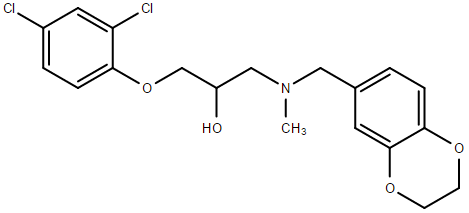  *(2R)-1-(2,4-dichlorophenoxy)-3-[2,3-dihydro-1,4-benzodioxin-6-ylmethyl(methyl)amino]propan-2-ol* | 398,3 | -13,4 | 37 | 4.5 |
| C8  Z28853604 | | 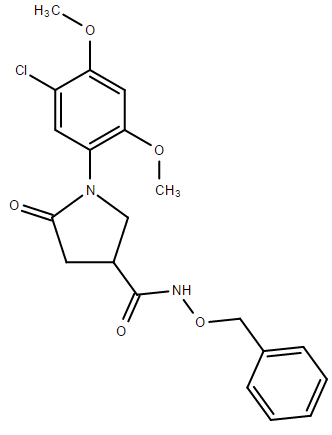  *(3S)-N-benzyloxy-1-(5-chloro-2,4-dimethoxy-phenyl)-5-oxo-pyrrolidine-3-carboxamide* | 404,8 | -14,2 | 247 | 4.12 |
| C9  Z65211380 | | 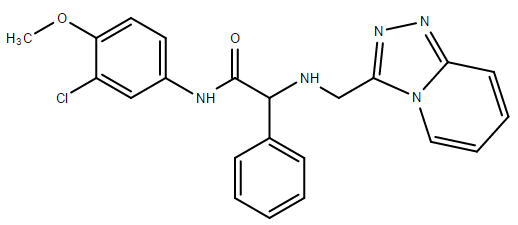  *(2R)-N-(3-chloro-4-methoxy-phenyl)-2-phenyl-2-([1,2,4]triazolo[4,3-a]pyridin-3-ylmethylamino)acetami* | 421,9 | -13,8 | 9 | 2.445 |
| C10  Z27304548 | | *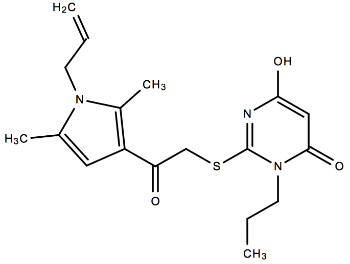*  *N-[5-chloro-2-(4-methylpiperazin-1-yl)-phenyl]-3-(2-fluorophenyl)sulfonylamino-propanamide* | 454,9 | -13,7 | 27 | ND |
| C11  Z46393258 | | 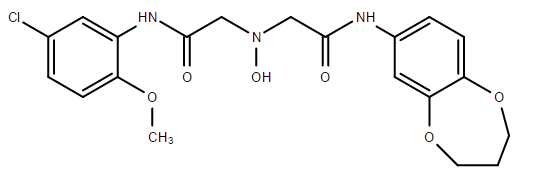  *2-[[2-[(5-chloro-2-methoxy-phenyl)amino]-2-oxo-ethyl]-methyl-amino]-N-(3,4-dihydro-2H-1,5-benzodioxe* | 433,9 | -13,9 | 33 | 2.949 |
| C12  Z106335952 | | 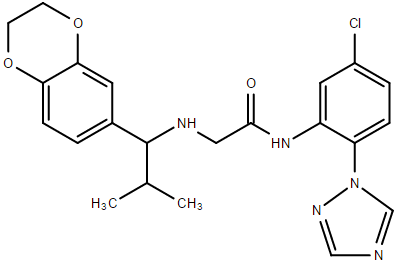  *N-[5-chloro-2-(1,2,4-triazol-1-yl)phenyl]-2-[[(1R)-1-(2,3-dihydro-1,4-benzodioxin-6-yl)-2-methyl-pro* | 441,9 | -14,0 | 76 | 3.791 |
| C13  Z235938578 | | 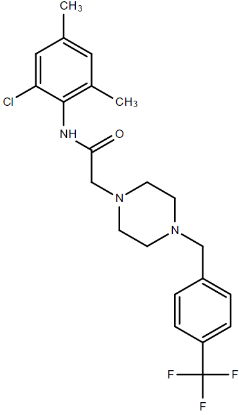  *N-(2-chloro-4,6-dimethyl-phenyl)-2-[4[[4-(trifluoromethyl)phenyl]methyl]-1,4-diazepan-1-yl]acetamid* | 453,9 | -14,0 | 413 | 4.859 |
| C14  Z105700580 | | 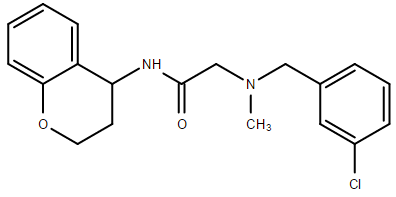  *2-[(3-chlorophenyl)methyl-methyl-amino]-N- chroman-4-yl-acetamide* | 344,8 | -14,7 | 29 | 3.634 |
| C15  Z52736477 | | 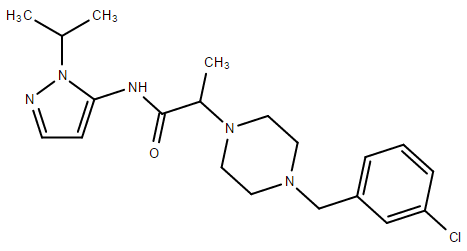  *(2R)-2-[4-[(3-chlorophenyl)methyl]piperazin-1-yl]-N-(2-isopropylpyrazol-3-yl)propanamide* | 389,9 | -15,9 | 342 | 3.957 |
| C16  Z202373826 | | 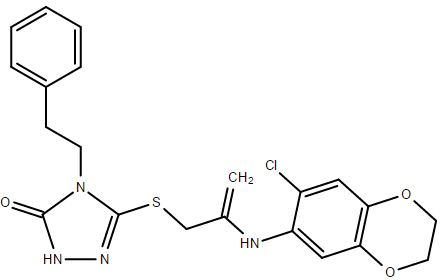  *N-(8-chloro-2,5-dioxabicyclo[4.4.0]deca-6,8,10-trien-9-yl)-2-[(5-oxo-4-phenethyl-1H-1,2,4-triazol* | 446,9 | -15,9 | 464 | 2.997 |
| C17  Z46406207 | | 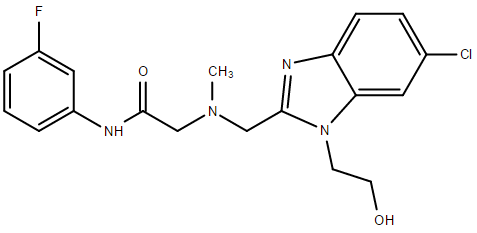  *2-[[6-chloro-1-(2-hydroxyethyl)benzomidazol-2-yl]methyl-methyl-amino]-N-(3-fluorophenyl)-acetamide* | 390,8 | -14,9 | 32 | 2.78 |
| C18  Z106459694 | | 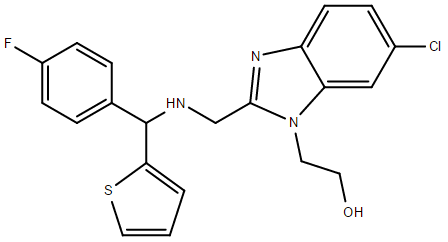  *2-[6-chloro-2-[[[(S)-(4-fluorophenyl)-(2-thienyl)methyl]amino]methyl]benzinidazol-1-yl]ethanol* | 415,9 | -15,2 | 34 | 3.153 |
| C19  Z44521301 | | 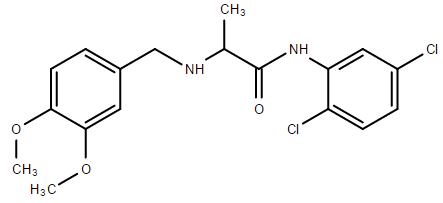  *N-(2,5-dichlorophenyl)-2-{[(3,4-dimethoxyphenyl)methyl]amino}propanamide* | 383,3 | -14,9 | 50 | 6.363 |
| C20  Z232357168 | | 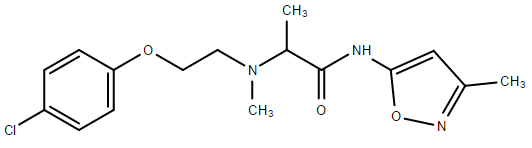  *2-[2-(4-chlorophenoxy)ethyl-methyl-amino]-N-(3-methylisoxazol-5-yl)-propanamide* | 337,8 | -14,3 | 349 | 3.554 |
| C21  Z126042658 | | *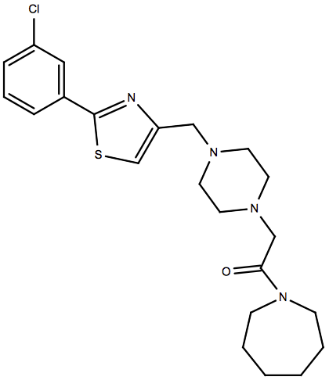*  *1-(azepan-1-yl)-2-[4-[[2-(4-chlorophenyl)thiazol-4-yl]methyl]piperzin-1-yl]ethanone* | 433 | -15,3 | 243 | 4.72 |
| C22  Z199813680 | | 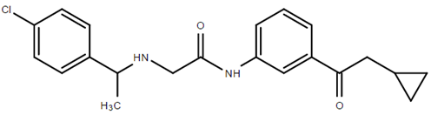  *3-[[2-[[(1R)-1-(4-chlorophenyl)ethyl]amino]acetyl]amino]-N-cyclopropyl-benzamide* | 371,9 | -13,8 | 64 | 3.091 |
| C23  Z89124359 | 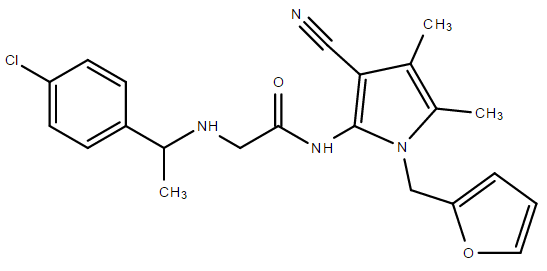  *2-[[(1S)-1-(4-chlorophenyl)ethyl]amino]-N-[3cyano-1-(2furymethyl)-4,5-dimethyl-pyrrol-2-yl]acetam* | | 410,9 | -14,3 | 95 | 3.815 |
| C24  Z52853441 | 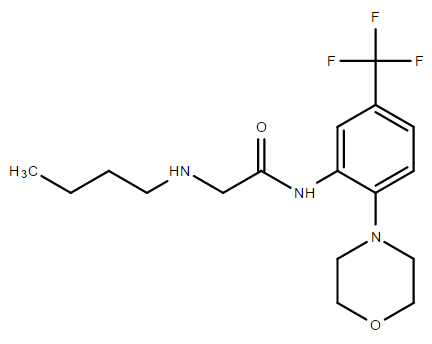  *Butyl-[2-keto-2-[2-morpholino-5 (trifluoromethyl)anilino]ethyl]ammonium* | | 359,4 | -13,5 | 8 | 3.058 |
