# Supplementary material for: KRas4B-PDE6δ complex stabilization by small molecules obtained by virtual screening affects Ras signaling in pancreatic cancer
Source: BMC Cancer. 2018 Dec 29;18:1299. doi: 10.1186/s12885-018-5142-7 (PMC6310981; doi:10.1186/s12885-018-5142-7)
Supplement: Supplementary file 2 — Table S2. The time-dependence of RMSD and RG by D14 and C22 compounds that target wild-type and mutated KRas4B-PDE6δ molecular complexes. (DOC 35 kb) [file 12885_2018_5142_MOESM2_ESM.doc]

**Table S2**. The time-dependence of RMSD and RG by D14 and C22 compounds that target wild-type and mutated KRas4B-PDE6δ molecular complexes.

| System | RMSD (Å) | RG (Å) |
| --- | --- | --- |
| Free and bound wild type KRas4B-PDE6δ complex | | |
| KRas4B-PDE6δ | 2.6 ± 0.3 | 22.5 ± 0.2 |
| KRas4B-PDE6δ-D14 | 2.7 ± 0.3 | 22.4 ± 0.2 |
| KRas4B-PDE6δ-C22 | 3.5 ± 0.6 | 22.4 ± 0.3 |
| Free and bound mutated KRas4BG12C-PDE6δ | | |
| KRas4BG12C-PDE6δ | 3.3 ± 0.3 | 22.2 ± 0.1 |
| KRas4BG12C -PDE6δ-D14 | 4.2 ± 0.4 | 22.6 ± 0.3 |
| KRas4BG12C-PDE6δ-C22 | 3.4 ± 0.4 | 22.8 ± 0.2 |
| Protein-ligand systems | | |
| PDE6δ-C22 | 1.9 ± 0.3 | 16.4 ± 0.1 |
| PDE6δ-D14 | 1.3 ± 0.2 | 16.2 ± 0.1 |

Average geometrical values (Å) over the last 50 ns of 100-ns-long MD simulations (± standard deviation).
