# Supplementary material for: KRas4B-PDE6δ complex stabilization by small molecules obtained by virtual screening affects Ras signaling in pancreatic cancer
Source: BMC Cancer. 2018 Dec 29;18:1299. doi: 10.1186/s12885-018-5142-7 (PMC6310981; doi:10.1186/s12885-018-5142-7)
Supplement: Supplementary file 3 — Table S3. Summary of histopathological analysis and in vivo evaluation by the D14 and C22 compounds on tumor growth in nude mice. (DOC 30 kb) [file 12885_2018_5142_MOESM3_ESM.doc]

| **Table S3**. Summary of histopathological analysis of *In vivo* evaluation of the effect of compounds D14 and C22 on tumor growth in nude mice. | | | | |
| --- | --- | --- | --- | --- |
| **Treatment** | **Description** | **Ki-67** | **Ck 19** |  |
| **Vehicle** | Neoplasm of poorly differentiated malignant epithelial lineage, constituted by cells with loss of the nucleus citomplasma relation, most of the nuclei present "open face", some pleomorphic, focal apoptotic figures. Necrosis in 5% of the tissue analyzed. 70% of tumor cells. A focus of inflammatory cells of chronic peritumoral type is identified. No residual tissue is identified. It identifies scarce adipose and muscle tissue without alterations | 10% +++, cel neopl. Nucleus, 5% ++, cel neop. Cytoplasm | 15% +++, cel neopl. Cytoplasm |  |
| **D14** | Neoplasm of poorly differentiated epithelial malignant lineage, constituted by cell nests with loss of the nucleus cytoplasm ratio, medians, apoptotic figures, 15% necrosis,. 40% of neoplastic cells. Adipose tissue without alterations. No residual tissue is identified. | 7% +++, cel neopl. Nucleus, 1% ++, cel neop. Cytoplasm cells | 15% +++, cel neopl. Cytoplasm |  |
| **C22** | Neoplasm of poorly differentiated epithelial malignant lineage, constituted by cell nests with loss of the nucleus cytoplasm ratio, medians, apoptotic figures, 5% necrosis,. 30% of neoplastic cells. Adipose tissue without alterations. No residual tissue is identified. | 3% +++, cel neopl. Nucleus, 5% ++, cel neop. Cytoplasm | 15% +++, cel neopl. Cytoplasm |  |
